# Synthesis of 2,4-Diaminopyrimidine Core-Based Derivatives and Biological Evaluation of Their Anti-Tubercular Activities

**DOI:** 10.3390/molecules22101592

**Published:** 2017-09-22

**Authors:** Yifan Ouyang, Hao Yang, Peng Zhang, Yu Wang, Sargit Kaur, Xuanli Zhu, Zhe Wang, Yutong Sun, Wei Hong, Yun Fong Ngeow, Hao Wang

**Affiliations:** 1 School of Pharmacy, Ningxia Medical University, Yinchuan 750004, China; ouyang_pharmacy@163.com (Y.O.); yhao246@163.com (H.Y.); zpzzjzp@163.com (P.Z.); wangyuxingfu@163.com (Y.W.); jf18700994479@163.com (X.Z.); 13239935127@163.com (Z.W.); syt931115@163.com (Y.S.); 2 Department of Pre-Clinical Sciences, Faculty of Medicine and Health Sciences, Universiti Tunku Abdul Rahman, Sungai Long campus, Kajang 43000, Selangor, Malaysia; sargitk3@gmail.com; 3 School of Chemistry and Chemical Engineering, North Minzu University, Yinchuan 750021, China; 4 KeyLaboratory of Hui Ethnic Medicine Modernization, Ministry of Education, Ningxia Medical University, Yinchuan 750004, China

**Keywords:** anti-tuberculosis, 2,4-diaminopyrimidine derivatives, synthesis

## Abstract

Tuberculosis (TB) is a chronic, potentially fatal disease caused by *Mycobacterium tuberculosis* (*Mtb*). The dihyrofolate reductase in *Mtb* (*mt*-DHFR) is believed to be an important drug target in anti-TB drug development. This enzyme contains a glycerol (GOL) binding site, which is assumed to be a useful site to improve the selectivity towards human dihyrofolate reductase (*h*-DHFR). There have been previous attempts to design drugs targeting the GOL binding site, but the designed compounds contain a hydrophilic group, which may prevent the compounds from crossing the cell wall of *Mtb* to function at the whole cell level. In the current study, we designed and synthesized a series of *mt*-DHFR inhibitors that contain a 2,4-diaminopyrimidine core with side chains to occupy the glycerol binding site with proper hydrophilicity for cell entry, and tested their anti-tubercular activity against *Mtb* H37Ra. Among them, compound **16l** showed a good anti-TB activity (MIC = 6.25 μg/mL) with a significant selectivity against vero cells. In the molecular simulations performed to understand the binding poses of the compounds, it was noticed that only side chains of a certain size can occupy the glycerol binding site. In summary, the novel synthesized compounds with appropriate side chains, hydrophobicity and selectivity could be important lead compounds for future optimization towards the development of future anti-TB drugs that can be used as monotherapy or in combination with other anti-TB drugs or antibiotics. These compounds can also provide much information for further studies on *mt*-DHFR. However, the enzyme target of the compounds still needs to be confirmed by pure *mt*-DHFR binding assays.

## 1. Introduction

There is an urgent need to develop new drugs for the treatment of tuberculosis (TB), a chronic disabling infection caused by *Mycobacterium tuberculosis* (*Mtb*). This pathogen has developed resistance to standard first- and second-line anti-TB drugs, leaving very few options for effective therapy. *para*-Aminosalicylic acid (PAS) is a key anti-TB drug that has been in use for over 60 years. Its anti-mycobacterial mechanism was not clearly understood until recently, when it was reported to be the pro-drug of an inhibitor of the *Mtb* dihyrofolate reductase (*mt*-DHFR) [1]. *mt*-DHFR catalyzes the reduction of dihydrofolate to tetrahydrolate in the folate metabolic pathway that leads to the synthesis of purines, pyrimidines and other proteins. Inhibition of the enzyme would cause cell death via the inhibition of DNA synthesis. This association of PAS with *mt*-DHFR inhibition encouraged scientists to focus once again on *mt*-DHFR as a potential target for anti-TB drugs.

**Figure 1:**
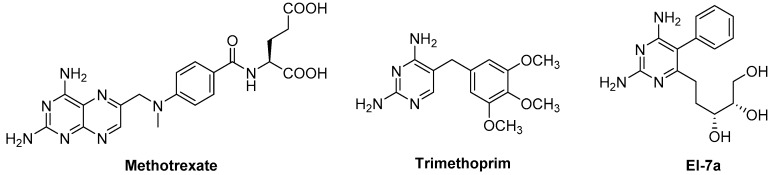
The structures of “classical” and “non-classical” DHFR inhibitors.

**Figure 2:**
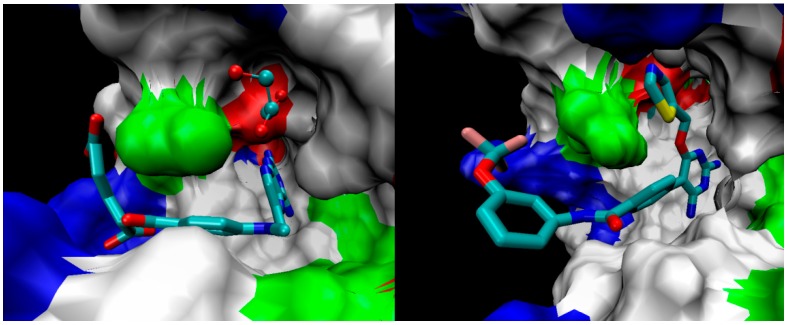
**Left:** the binding sites of methotrexate (MTX) and glycerol (GOL) in *mt*-DHFR, in which MTX is represented as a sticks model, GOL as a ball-stick model, and protein as a molecular surface; **Right:** the designed molecule is predicted to be able to occupy the GOL binding site, in which the molecule is represented as sticks and protein as a molecular surface.

According to their chemical structures, DHFR inhibitors can be divided into “classical” and “non-classical” types [2,3]. The structures of classical inhibitors are similar to that of folate, as in methotrexate (Figure 1), which is a commonly used anticancer drug [4,5,6]. The core structure of non-classical inhibitors is 2,4-diaminopyrimidine, as in trimethoprim (Figure 1), an anti-bacterial drug [7,8,9]. The crystal structures of *mt*-DHFR (PDB ID: 1DF7, Figure 2) and human DHFR (*h*-DHFR, PDB ID: 1OHJ), show a glycerol (GOL) binding site in *mt*-DHFR that does not exist in *h*-DHFR. To take advantage of this difference, Threadgill evaluated a group of compounds containing glycerol-like side chains, among which one compound, **El-7a** (Figure 1) showed notable selectivity for *mt*-DHFR inhibition over *h*-DHFR [10]. However, this evaluation was conducted using TB5 *Saccharomyces cerevisiae* carrying *mt*-DHFR and *h*-DHFR genes, therefore, there is no direct evidence to show that **El-7a** can inhibit the growth of *Mtb*. Furthermore, the inhibition of *Mtb* may require an appropriate lipophilicity in the compound [11]. Even if **El-7a** were able to selectively inhibit the *mt*-DHFR, its low hydrophobicity may prevent it from passing through the *Mtb* cell wall. Hence, we designed and synthesized a series of compounds containing more hydrophobic groups on the 6-position of 2,4-diaminopyrimidine to evaluate the ability of these compounds to inhibit *Mtb* cells directly.

## 2. Results and Discussion

### 2.1. Chemistry

**Scheme 1:**
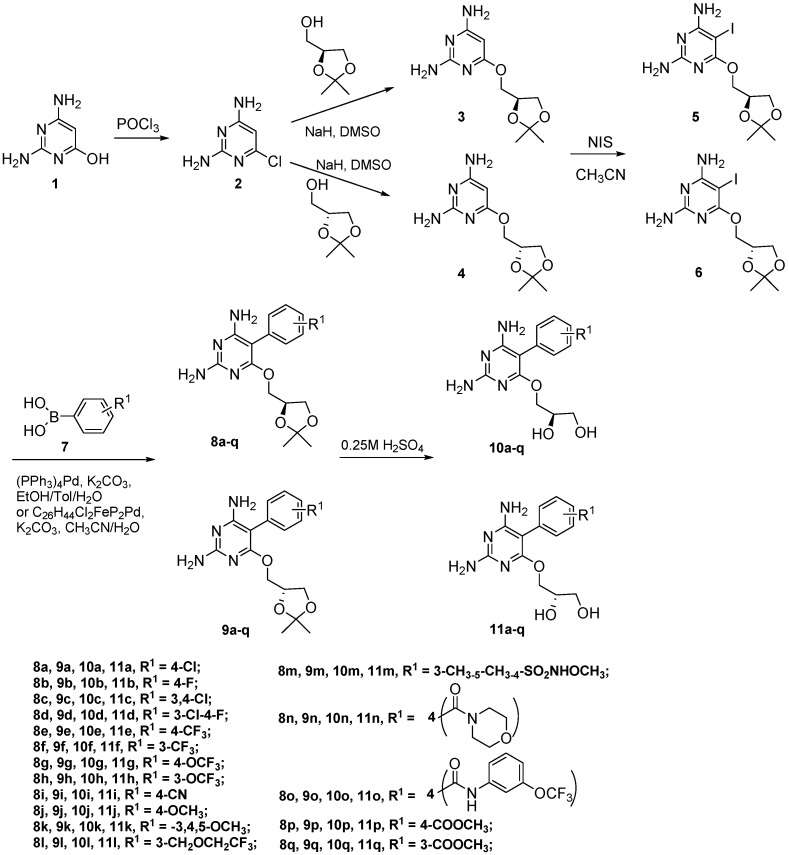
Synthesis of 2,4-diamino-5-aryl-6-substituted pyrimidine derivatives **10a-q** and **11a-q**.

The synthesis of the designed compounds **10a-q** or **11a-q** was carried out in five steps—chlorination, nucleophilic substitution, iodination, Suzuki reaction and deprotection—according to our patented method [12], with 2,4-diamino-6-hydroxypyrimidine (**1**) as the starting material (Scheme 1). Initially, 2,4-diamino-6-chloropyrimidine (**2**) was generated from **1** by treatment with phosphorus oxychloride. After the reaction was quenched with ice water, the solution was hydrolyzed at 90 °C to obtain a good yield (85%) of the target intermediate [13]. This procedure yields pure **2** without the need for chromatography.

The treatment of (*S*)-2,3-isopropylidene glycerol or (*R*)-2,3-isopropylidene glycerol with sodium hydride in dry DMSO generated the corresponding nucleophile, which was then reacted with **2** to give 2,4-diamino-6-substituted pyrimidines **3** or **4** in good yield (77%) [14]. Subsequently, the 5-positions of **3** or **4** were iodinated with *N*-iodosuccinimide in dry acetonitrile to produce the precursor 2,4-diamino-5-iodo-6-substituted pyrimidine derivatives **5** or **6** in 96–98% yields [15]. Initially, we used a previously synthesized 2,4-diamino-5-bromo-6-substituted pyrimidine derivative as the starting material in the model reaction of the Suzuki reactions in order to introduce a substituted aryl group in the 5-position of the 2,4-diaminopyrimidine core. We tried several conditions, such as reacting with phenylboronic acid, 4-chlorophenylboronic acid and 4-methoxycarbonylphenylboronic acid, different catalysts (Pd(PPh_3_)_4_ [16], Pd(dbpf)Cl_2_) [17], different bases (K_2_CO_3_, K_2_HPO_4_), different solvents (EtOH/Toluene/H_2_O, THF/H_2_O, CH_3_CN/H_2_O, Dimethoxyethane/H_2_O), different temperatures (70, 80, 90, 120 °C), and different heating modes (microwave or oil bath). Unfortunately, all these reactions failed. Subsequently, we realized that the iodide replacement in the 5-position of the 2,4-diaminopyrimidine core in the Suzuki reaction might be easier than with a bromide. Hence, the iodides **5** or **6** were allowed to react with substituted phenylboronic acids **7** under Suzuki reaction conditions (Pd(PPh_3_)_4_ as catalyst, K_2_CO_3_ as base, and EtOH/toluene/H_2_O as solvent). However, the products **8a-o** or **9a-o** were only obtained in 52–78% yields. Moreover, the presence of an electron donating (OCH_3_) or electron withdrawing (F, CF_3_, OCF_3_, ester, amide) group on the substituted phenylboronic acid **7** did not show high effect of the reaction yields. However, when the products **8p-q** or **9p-q** were synthesized by reacting **5** or **6** with 4- or 3-methoxycarbonylphenylboronic acids **7p** or **7q** in EtOH/toluene/H_2_O, the transesterification product was detected in the ^1^H-NMR sprectrum. Hence, different Suzuki reaction conditions were chosen, in which **5** or **6** was reacted with **7p** or **7q** with Pd(dbpf)Cl_2_ as catalyst, K_2_CO_3_ as base and CH_3_CN/H_2_O as solvent, to give **8p-q** or **9p-q** with 63–78% yields. Subsequently, the compounds **8a-q** or **9a-q** were deprotected in 0.25 M H_2_SO_4_ solution to generate the target compounds **10a-q** or **11a-q** in 68–95% yields.

**Scheme 2:**
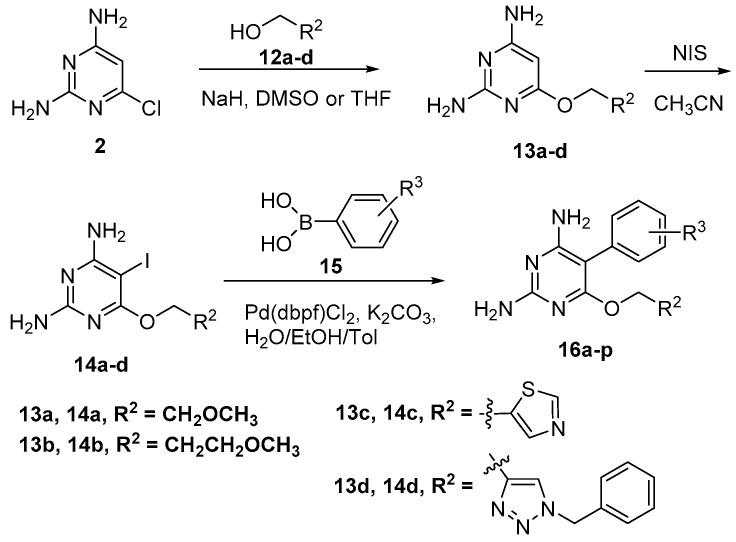
Synthesis of 2,4-diamino-5-aryl-6-substituted pyrimidine derivatives **16a-p**.

Subsequently 2,4-diamino-5-aryl-6-substituted pyrimidine derivatives **16a-p** were synthesized from 2,4-diamino-6-chloropyrimidine (**2**, Scheme 2) following our patented approach [18]. The treatment of substituted methanols **12a-d** with sodium hydride in dry DMSO or THF generated the corresponding nucleophiles, which were reacted with **2** to give 2,4-diamino-6-substituted pyrimidines **13a-d** in moderate to good yields (61–79%) [14]. Subsequently, the 5-positions of **13a-d** were iodinated with *N*-iodosuccinimide in dry acetonitrile to produce the precursor 2,4-diamino-5-iodo-6-substituted pyrimidine derivatives **14a-d** [15], which were used in subsequent Suzuki reactions to generate the target compounds **16a-p** in good yields (70–92%) [17].

A series of reactions in which iodides **14a-d** were used as staring material, was investigated as shown in Table 1. The iodides **14a-d** were reacted with substituted phenylboronic acid **15** in the presence of Pd(dbpf)Cl_2_ and K_2_CO_3_ in EtOH/toluene/H_2_O at 90 °C for 24 h (Table 1, Entries 1–14) or in the presence of Pd(dbpf)Cl_2_ and K_2_CO_3_ in THF/H_2_O at 70 °C for 20 h in a sealed tube (Table 1, Entries 15, 16) to generate the desired compounds **16a-p** with moderate to good yields (51–99%). For the iodide **14a**, treatment with different substituted phenylboronic acids **15** (Table 1, Entries 1–5) resulted in similar yields. For the iodide **14b**, reaction with **15** bearing a 3-trifloromethoxy group resulted in higher yields (Table 1, Entry 7) than when **15** bore a 4-trifloromethoxy group (Table 1, Entry 6). For the iodide **14c**, treatment with **15** substituted with a 3-trifloromethoxyanilinocarbonyl group or 3-(2,2,2-trifloroethoxymethyl) group (Table 1, Entries 10, 12) resulted in higher yields than with 4-trifloromethoxy, 3-trifloromethoxy and 3,5-dimethyl-4-(*N*-methoxyaminosulfonyl) groups substituted on **15** (Table 1, Entries 8, 9, 11). Especially for the iodide **14d**, reaction with **15** bearing a 4-trifloromethoxy or 3-trifloromethoxy group (Table 1, Entries 13, 14) resulted in much higher yields than with **15** bearing a 4-methoxycarbonyl or 3-methoxycarbonyl group (Table 1, Entries 15, 16). The presumption is that the ester group on this Suzuki reaction could be affected by transesterification in the solvent (EtOH/toluene/H_2_O), which could be detected in ^1^H-NMR. Thus, in these reactions, we chose THF/H_2_O as the solvent to avoid the transesterification.

### 2.2. Determination of In Vitro Anti-Tubercular Activity

Based on the different R^2^ substituents on the 2,4-diamino-5-aryl-6-substituted pyrimidine derivatives, the compounds can be divided into four types: (1) the R^2^ substituents bearing hydroxy groups (**10a-q**, **11a-q**); (2) the R^2^ substituents bearing alkoxy groups (**16a-g**); (3) the R^2^ substituents bearing thiazole groups (**16h-l**); (4) the R^2^ substituents bearing phenyl substituted triazole groups (**16m-p**). Only compounds containing the thiazole group act as *Mtb* inhibitors. Among this group of compounds, five compounds (**16h-l**) showed potentially useful inhibitory effects, with **16l** showing the lowest MIC (6.25 μg/mL or 12.45 μM) and MBC (12.5 μg/mL) (Table 2). In order to see the selectivity of **16l** against mammalian cells, the MTT assay was performed on vero cells, and the IC_50_ on cells viability was found to be around 50.22 μM. The selectivity ratio of **16l** on H37Ra vs vero cells is around 4-fold.

### 2.3. Molecular Docking and Simulation

**Figure 3:**
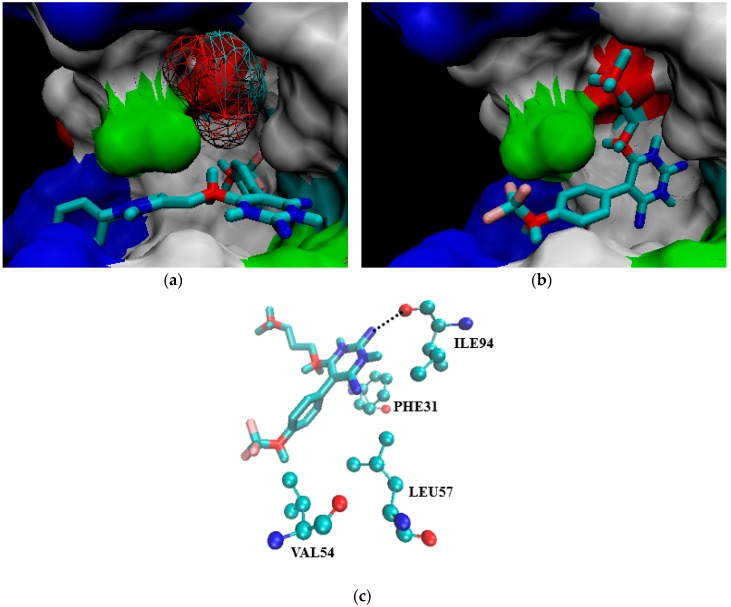
(**a**) Compound **16m** with large side chain group and the binding sites of glycerol (GOL) in *mt*-DHFR, in which compound **16m** is represented as a sticks model, GOL as a dotted line model and protein as a molecular surface; (**b**,**c**) the small side chain group of compound **16f** can fit into the GOL binding site, in which the molecule is represented as sticks and protein as a molecular surface.

Through the structural analysis of the compounds, the Clog P of **El-7a** was noticed to be −0.17, which showed the compound **El-7a** to be very hydrophilic, and led us to assume it would not be able to cross the *Mtb* cell wall. This assumption was indirectly confirmed by the observation that the hydrophilic compounds **10a-q** and **11a-q** (analogs of **El-7a**, with Clog P around −1 to 2), could not inhibit the growth of *Mtb*. Based on the above assumption, more hydrophobic compounds were analyzed by using molecular docking and molecular dynamic simulations, and based on the size of the substituents on the 6-position of 2,4-diaminopyrimidine, they were divided into three groups, which are large side chain groups (compounds **16m-p**), medium side chain groups (compounds **16h-l**) and small side chain groups (compounds **16a-g**). With molecular docking, it was noticed that the large side chain group, which contains the 1-benzyl-1*H*-1,2,3-triazole-4-methoxy group on the 6-position, cannot fit into the GOL binding site (Figure 3a), and this could be the reason why this group of compounds did not show any inhibition effects on *Mtb*. Although the small side chain group derivatives (compounds **16a-g**), which contain the methoxyethoxy or methoxypropoxy group on the 6-positions, can fit into the GOL binding site (Figure 3b,c), they cannot form strong interactions or fully fill the GOL binding site.

**Figure 4:**
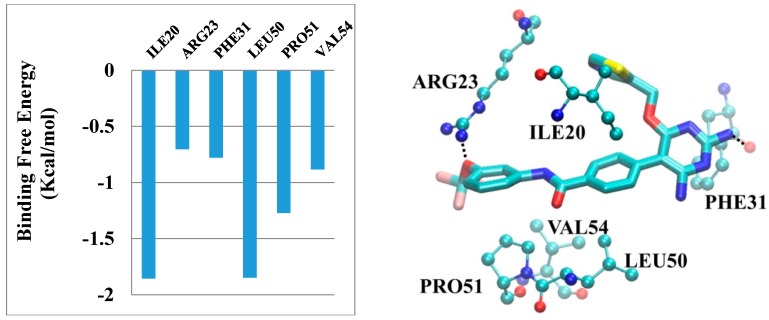
**Left**: The residues whose binding free energy contributions are greater than −0.5 Kcal/mol; **Right**: The interactions between key residues and compound **16l**.

The molecular docking showed that the medium side chain group (compounds **16h-l**), which contain a (thiazol-5-yl)methoxy on the 6-position, can fit into the GOL binding site properly, and a molecular dynamics simulation was performed to understand the binding of compound **16l** to *mt*-DHFR. During 100 ns simulations, **16l** was stable in the binding site, and the side chain of compounds **16h-l** occupied the GOL binding site along the full simulation (Figure 2). The free energy calculation showed that the binding free energy was −3.47 Kcal/mol (Table 3), which indicated that **16l** can bind with *mt*-DHFR tightly. The free energy contributions of each residue was calculated, and contributions greater than −0.5 Kcal/mol were recorded (Ile20, Arg23, Phe31, Leu50, Pro51 and Val54) (Figure 4 Left). Most of these residues showed a strong VDW interaction, except Phe31 and Arg23 which formed a H-bond with **16l**. Arg23 could also form strong interactions with the trifluoromethoxy group (Figure 4 Right).

Therefore, through the molecular docking and molecular dynamic simulations, we believe that the compounds containing the (thiazol-5-yl)methoxy side chain (medium size group), can fully occupy the GOL binding site, and have reasonable properties. Therefore, such compounds could be used as the lead compounds for further anti-TB drug discovery studies.

## 3. Materials and Methods

### 3.1. General Information

All reagents and solvents were purchased from the suppliers and used directly in the experiments. THF was dried by distillation ver sodium benzophenone. TLC was carried out using silica gel 60 pre-coated aluminium plates (0.20 mm thickness) from Macherey-Nagel (Darmstadt, Germany) with visualisation by UV light (254 nm). Flash chromatography was performed on silica gel (particle size 40–63 μm). IR spectra were recorded on a Tensor 27 spectrometer (Bruker, Ettlingen, Germany) using KBr discs. ^1^H-NMR spectra were obtained from an AVANCE III 400 spectrometer (Bruker, Fällanden Switzerland). The chemical shifts, given as δ values, were quoted in parts per million (ppm); ^1^H-NMR chemical shifts were measured relative to internal tetramethylsilane; Apparent coupling constants (absolute values), *J*, were measured in Hertz and multiplicities quoted as singlet (s), doublet (d), triplet (t), quartet (q) or combinations thereof as appropriate. Mass spectra were obtained from an 6545 Accurate-Mass Q-TOF LC/MS (Agilent Technologies, Santa Clara, CA, USA). Melting points were determined using a WRS-1B melting point measurement instrument (Shanghai, China) and were uncorrected. 

### 3.2. ChemistryIt 

*2,4-Diamino-6-chloropyrimidine* (**2**). 2,4-Diamino-6-hydroxypyrimidine (**1**) (1.00 g, 7.93 mmol) was added to POCl_3_ (9 mL), and stirred at 97 °C for 17 h. The reaction solution was added to ice water slowly, and then stirred at 90 °C for 1 h. The pH of this solution was adjusted to 8 with NaOH, and then it was extracted with EtOAC (150 mL × 3). The combined organic layers were dried with Na_2_SO_4_, filtered and concentrated to give white solid 0.97 g, yield 85%. m.p. 200.2–200.4 °C; IR (KBr): υ_max_/cm^−1^ 3449 (NH), 3327 (NH), 1642 (C=N), 1581 (C=C), 1551 (C=C), 795 (C-Cl); ^1^H-NMR (DMSO-*d*_6_) δ 6.57 (s, 2H, N*H*_2_), 6.31 (s, 2H, N*H*_2_), 5.69 (s, 1H, Ar-*H*); ES-MS 145.0 (M + H)^+^; HRMS Calcd. for C_16_H_20_ClN_4_O_3_^+^ 145.0281, found 145.0276.

#### General Procedure for the Synthesis of Compounds **3** and **4**

Under argon, to a solution of (*S*)-2,3-isopropylideneglycerol or (*R*)-2,3-isopropylideneglycerol 0.50 mL (4.0 mmol) in dry DMSO (5 mL) was added NaH 0.20 g (60%, 5.0 mmol) and stirred at room temperature for 1 h. 2,4-Diamino-6-chloropyrimidine (**2**, 0.29 g, 2.0 mmol) was added and stirred at 90 °C for 8 h. The reaction solution was quenched with sat NH_4_Cl (20 mL) and extracted with EtOAc (30 mL × 3), and the combined organic layers dried with Na_2_SO_4_, filtered and concentrated. The residue was purified by column chromatography on silica gel using CH_2_Cl_2_/CH_3_OH (50:1, *v*/*v*) as the eluting solvent to give compounds **3** or **4**.

*(R)-2,4-Diamino-6-[4-(2,2-dimethyl-1,3-dioxolane)methoxy]pyrimidine* (**3**). White solid 0.37 g, yield 77%; m.p. 89.7–91.0 °C; IR: υ_max_/cm^−1^ 3474 (NH), 3452 (NH), 3373 (NH), 3359 (NH), 1668 (C=N), 1627 (C=N), 1593 (C=C), 1200 (C-O-C), 1157 (C-O-C), 1082 (C-O-C), 1052 (C-O-C); ^1^H-NMR (CDCl_3_) δ 5.28 (s, 1H, Ar-*H*), 4.71 (s, 2H, N*H*_2_), 4.53 (s, 2H, N*H*_2_), 4.41 (q, *J* = 6.0, 1H, C*H*), 4.25 (d, *J* = 5.6, 2H, OC*H*_2_), 4.11 (dd, *J*_1_ = 8.2, *J*_2_ = 6.4, 1H, OC*H*_2_), 3.81 (dd, *J*_1_ = 8.4, *J*_2_ = 6.4, 1H, OC*H*_2_), 1.44 (s, 3H, C*H*_3_), 1.38 (s, 3H, C*H*_3_); ES-MS 241.1 (M + H)^+^; HRMS Calcd. for C_16_H_20_ClN_4_O_3_^+^ 241.1301, found 241.1303.

*(S)-2,4-Diamino-6-[4-(2,2-dimethyl-1,3-dioxolane)methoxy]pyrimidine* (**4**). White solid 10.32 g, yield 77%; m.p. 87.6–88.9 °C; IR: υ_max_/cm^−1^ 3474 (NH), 3451 (NH), 3373 (NH), 3358 (NH), 1670 (C=N), 1626 (C=N), 1592 (C=C), 1199 (C-O-C), 1159 (C-O-C), 1085 (C-O-C), 1054 (C-O-C); ^1^H-NMR (CDCl_3_) δ 5.29 (s, 1H, Ar*-H*), 4.67 (s, 2H, N*H*_2_), 4.50(s, 2H, N*H*_2_), 4.41 (q, *J* = 6.0, 1H, C*H*), 4.29–4.22 (m, 2H, OC*H*_2_), 4.11 (dd, *J*_1_ = 8.8, *J*_2_ = 6.4, 1H, OC*H*_2_), 3.82 (dd, *J*_1_ = 8.4, *J*_2_ = 6.0, 1H, OC*H*_2_), 1.45 (s, 3H, C*H*_3_), 1.38 (s, 3H, C*H*_3_); ES-MS 241.1 (M + H)^+^; HRMS Calcd. for C_16_H_20_ClN_4_O_3_^+^ 241.1301, found 241.1306.

#### General Procedure for the Synthesis of Compounds **5** and **6**

Under argon, to a solution of **3** or **4** (7.18 g, 29.88 mmol) in dry CH_3_CN (100 mL) was added *N*-iodosuccinimide 10.09 g (44.83 mmol) and stirred at room temperature for 1 h. The reaction solution was diluted with EtOAc (500 mL), washed by 5% NaHSO_3_ (500 mL), NaHCO_3_ (500 mL) and H_2_O (500 mL), and dried with Na_2_SO_4_, filtered and concentrated. The residue was purified by column chromatography on silica gel using CH_2_Cl_2_/CH_3_OH (100:1, *v*/*v*) as the eluting solvent to give compounds **5** or **6**.

*(R)-2,4-Diamino-5-iodo-6-[4-(2,2-dimethyl-1,3-dioxolane)methoxy]pyrimidine* (**5**). White solid 13.42 g, yield 98%; m.p. 135.5–136.8 °C; IR: υ_max_/cm^−1^ 3464 (NH), 3402 (NH), 3356 (NH), 1649 (C=N), 1627 (C=N), 1550 (C=C), 1200 (C-O-C), 1157 (C-O-C), 1082 (C-O-C), 1052 (C-O-C), 476 (C-I); ^1^H-NMR (CDCl_3_) δ 5.06 (s, 2H, N*H*_2_), 4.69 (s, 2H, N*H*_2_), 4.44–4.37 (m, 2H, OC*H*_2_ and C*H*), 4.30–4.25 (m, 1H, OC*H*_2_), 4.12 (dd, *J*_1_ = 8.4, *J*_2_ = 6.4, 1H, OC*H*_2_), 3.96 (dd, *J*_1_ = 8.4, *J*_2_ = 6.0, 1H, OC*H*_2_), 1.47 (s, 3H, C*H*_3_), 1.39 (s, 3H, C*H*_3_); ES-MS 367.0 (M + H)^+^; HRMS Calcd. for C_16_H_20_ClN_4_O_3_^+^ 367.0267, found 367.0265.

*(S)-2,4-Diamino-5-iodo-6-[4-(2,2-dimethyl-1,3-dioxolane)methoxy]pyrimidine* (**6**). White solid 10.72 g, yield 96%; m.p. 135.4–136.7 °C; IR: υ_max_/cm^−1^ 3465 (NH), 3401 (NH), 3360 (NH), 3310 (NH), 1650 (C=N), 1623 (C=N), 1549 (C=C), 1200 (C-O-C), 1157 (C-O-C), 1133 (C-O-C), 1096 (C-O-C), 1071 (C-O-C), 1045 (C-O-C), 478 (C-I); ^1^H-NMR (CDCl_3_) δ 5.05 (s, 2H, N*H*_2_), 4.68 (s, 2H, N*H*_2_), 4.37–4.34 (m, 2H, OC*H*_2_ and C*H*), 4.30–4.25 (m, 1H, C*H*_2_), 4.12 (dd, *J*_1_ = 8.4, *J*_2_ = 6.4, 1H, OC*H*_2_), 3.96 (dd, *J*_1_ = 8.4, *J*_2_ = 6.0, 1H, OC*H*_2_), 1.47 (s, 3H, C*H*_3_), 1.39 (s, 3H, C*H*_3_); ES-MS 367.0 (M + H)^+^; HRMS Calcd. for C_16_H_20_ClN_4_O_3_^+^ 367.0267, found 367.0268.

#### General Procedure for the Synthesis of Compounds **8a-q** and **9a-q**

(A) Under argon, to a mixed solution of EtOH/toluene (1:2, 60 mL) was added compound **5** or **6** (2.73 mmol), substituted phenylboronic acid (**7**) (3.00–5.46 mmol), Pd(PPh_3_)_4_ (1.38 × 10^−4^ mmol) and K_2_CO_3_ (3 M, 3.00–5.50 mL) consecutively and then stirred at 90 °C for 1–2 h. The reaction solution was extracted with EtOAc (30 mL × 3), and the combined organic layers were washed by H_2_O and dried with Na_2_SO_4_, filtered and concentrated. The residue was purified by column chromatography on silica gel using CH_2_Cl_2_/CH_3_OH (80:1, *v*/*v*) as the eluting solvent to the desired compounds.

(B) In a pressure tube, to a mixed solution of CH_3_CN/H_2_O (1:1, 40 mL) was added compound **5** or **6** (2.73 mmol), substituted phenylboronic acid **7p** or **7q** (4.10 mmol), Pd(dbpf)Cl_2_ (2.73 × 10^−4^ mmol) and K_2_CO_3_ (4.10 mmol) consecutively and then stirred at 60 °C for 8 h. The reaction solution was extracted with EtOAc (30 mL × 3), and the combined organic layers were washed by H_2_O and dried with Na_2_SO_4_, filtered and concentrated. The residue was purified by column chromatography on silica gel using CH_2_Cl_2_/CH_3_OH (60:1, *v*/*v*) as the eluting solvent to the desired compounds.

*(R)-2,4-Diamino-5-(4-chlorophenyl)-6-[4-(2,2-dimethyl-1,3-dioxolane)methoxy]pyrimidine* (**8a**). Method A. White solid 0.64 g, yield 67%; m.p. 171.2–172.8 °C; IR: υ_max_/cm^−1^ 3475 (NH), 3454 (NH), 3305 (NH), 3315 (NH), 1649 (C=N), 1620 (C=N), 1592 (C=C), 1549 (C=C), 1479 (C=C), 1160 (C-O-C), 1144 (C-O-C), 1089 (C-O-C), 794 (C-Cl); ^1^H-NMR (CDCl_3_) δ 7.36 (d, *J* = 8.4, 2H, Ar-*H*), 7.25 (d, *J* = 8.0, 2H, Ar-*H*), 4.69 (s, 2H, N*H*_2_), 4.52 (s, 2H, N*H*_2_), 4.36 (dd, *J*_1_ = 10.8, *J*_2_ = 4.4, 1H, OC*H*_2_), 4.31–4.26 (m, 1H, CH), 4.20 (dd, *J*_1_ = 10.8, *J*_2_ = 5.6, 1H, OC*H*_2_), 3.97 (dd, *J*_1_ = 8.4, *J*_2_ = 6.4, 1H, OC*H*_2_), 3.72 (dd, *J*_1_ = 8.4, *J*_2_ = 6.0, 1H, OC*H*_2_), 1.32 (s, 3H, C*H*_3_), 1.28 (s, 3H, C*H*_3_); ES-MS 351.1 (M + H)^+^; HRMS Calcd. for C_16_H_20_ClN_4_O_3_^+^ 351.1224, found 351.1222.

*(R)-2,4-Diamino-5-(4-fluorophenyl)-6-[4-(2,2-dimethyl-1,3-dioxolane)methoxy]pyrimidine* (**8b**). Method A. White solid 0.61 g, yield 74%; m.p. 162.3–163.0 °C; IR: υ_max_/cm^−1^ 3526 (NH), 3483 (NH), 3369 (NH), 3312 (NH), 1647 (C=N), 1626 (C=N), 1593 (C=C), 1558 (C=C), 1510 (C=C), 1485(C=C), 1383 (C-F), 1160 (C-O-C), 1128 (C-O-C), 1105 (C-O-C), 1090 (C-O-C); ^1^H-NMR (CDCl_3_) δ 7.27 (dd, *J*_1_ = 8.8, *J*_2_ = 5.6, 2H, Ar-*H*), 7.08 (t, *J* = 8.8, 2H, Ar-*H*), 4.69 (s, 2H, N*H*_2_), 4.52 (s, 2H, N*H*_2_), 4.36 (dd, *J*_1_ = 10.8, *J*_2_ = 4.0, 1H, OC*H*_2_), 4.31–4.26 (m, 1H, C*H*), 4.20 (dd, *J*_1_ = 10.8, *J*_2_ = 5.6, 1H, OC*H*_2_), 3.96 (dd, *J*_1_ = 8.4, *J*_2_ = 6.4, 1H, OC*H*_2_), 3.71 (dd, *J*_1_ = 8.4, *J*_2_ = 6.0, 1H, OC*H*_2_), 1.32 (s, 3H, C*H*_3_), 1.27 (s, 3H, C*H*_3_); ES-MS 335.2 (M + H)^+^; HRMS Calcd. for C_16_H_20_FN_4_O_3_^+^ 335.1519, found 335.1515.

*(R)-2,4-Diamino-5-(3,4-dichlorophenyl)-6-[4-(2,2-dimethyl-1,3-dioxolane)methoxy]pyrimidine* (**8c**). Method A. White solid 0.55 g, yield 52%; m.p. 164.1–165.9 °C; IR: υ_max_/cm^−1^ 3487 (NH), 3378 (NH), 3316 (NH), 1651 (C=N), 1624 (C=N), 1600 (C=C), 1556 (C=C), 1489 (C=C), 1154 (C-O-C), 1134 (C-O-C), 1191 (C-O-C), 1073 (C-O-C), 795 (C-Cl); ^1^H-NMR (CDCl_3_) δ 7.45 (d, *J* = 8.0, 1H, Ar-*H*), 7.44 (d, *J* = 2.0, 1H, Ar-*H*), 7.17 (dd, *J*_1_ = 8.0, *J*_2_ = 2.0, 1H, Ar-*H*), 4.73 (s, 2H, N*H*_2_), 4.56 (s, 2H, N*H*_2_), 4.35 (dd, *J*_1_ = 10.4, *J*_2_= 4.4, 1H, OC*H*_2_), 4.33–4.27 (m, 1H, C*H*), 4.22 (dd, *J*_1_ =10.4 , *J*_2_ = 5.2, 1H, OC*H*_2_), 3.99 (dd, *J*_1_ = 8.4, *J*_2_ = 6.0, 1H, OC*H*_2_), 3.73 (dd, *J*_1_ = 8.4, *J*_2_ = 6.0, 1H, OC*H*_2_), 1.33 (s, 3H, C*H*_3_), 1.30 (s, 3H, C*H*_3_); ES-MS 385.1 (M + H)^+^; HRMS Calcd. for C_16_H_19_Cl_2_N_4_O_3_^+^ 385.0834, found 385.0830.

*(R)-2,4-Diamino-5-(3-chloro-4-fluorophenyl)-6-[4-(2,2-dimethyl-1,3-dioxolane)methoxy]pyrimidine* (**8d**). Method A. White solid 0.64 g, yield 64%; m.p. 149.1–150.1 °C; IR: υ_max_/cm^−1^ 3484 (NH), 3382 (NH), 3320 (NH), 1650 (C=N), 1621(C=N), 1594 (C=C), 1553 (C=C), 1502 (C=C), 1372 (C-F), 1160 (C-O-C), 1092 (C-O-C), 1065 (C-O-C), 797 (C-Cl); ^1^H-NMR (CDCl_3_) δ 7.37 (dd, *J*_1_ = 7.2, *J*_2_ = 1.6, 1H, Ar-*H*), 7.20–7.14 (m, 2H, Ar-*H*), 4.71 (s, 2H, N*H*_2_), 4.53 (s, 2H, N*H*_2_), 4.35 (dd, *J*_1_ = 10.6, *J*_2_ = 4.2, 1H, OC*H*_2_), 4.32–4.27 (m, 1H, C*H*), 4.22 (dd, *J*_1_ = 10.6, *J*_2_ = 5.4, 1H, OC*H*_2_), 3.98 (dd, *J*_1_ = 8.4, *J*_2_ = 6.0, 1H, OC*H*_2_), 3.72 (dd, *J*_1_ = 8.4, *J*_2_ = 6.0, 1H, OC*H*_2_), 1.33 (s, 3H, C*H*_3_), 1.29 (s, 3H, C*H*_3_); ES-MS 369.1 (M + H)^+^; HRMS Calcd. for C_16_H_19_ClFN_4_O_3_^+^, 369.1130, found 369.1128.

*(R)-2,4-Diamino-5-(4-trifluoromethylphenyl)-6-[4-(2,2-dimethyl-1,3-dioxolane)methoxy]pyrimidine* (**8e**). Method A. Light yellow solid 0.72 g, yield 69%; m.p. 126.6–127.2 °C; IR: υ_max_/cm^−1^ 3530 (NH), 3511 (NH), 3444 (NH), 3420 (NH), 1649 (C=N), 1609 (C=N), 1593 (C=C), 1558 (C=C), 1491 (C=C), 1171 (C-O-), 1128 (C-O-C), 1094 (C-O-C), 1072 (C-O-C), 1046 (C-O-C); ^1^H-NMR (CDCl_3_) δ 7.64 (d, *J* = 8.2, 2H, Ar-*H*), 7.46 (d, *J* = 8.2, 2H Ar-*H*), 4.73 (s, 2H, N*H*_2_), 4.56 (s, 2H, N*H*_2_), 4.37 (dd, *J*_1_ = 11.2, *J*_2_ = 4.2, 1H, OC*H*_2_), 4.32–4.27 (m, 1H, C*H*), 4.22 (dd, *J*_1_ = 11.2, *J*_2_ = 5.6, 1H, OC*H*_2_*)*, 3.97 (dd, *J*_1_ = 8.4, *J*_2_ = 6.0, 1H, OC*H*_2_), 3.71 (dd, *J*_1_ = 8.4, *J*_2_ = 6.0, 1H, OC*H*_2_), 1.32 (s, 3H, C*H*_3_), 1.24 (s, 3H, C*H*_3_); ES-MS 385.1 (M + H)^+^; HRMS Calcd. for C_17_H_20_F_3_N_4_O_3_^+^ 385.1488, found 385.1485.

*(R)-2,4-Diamino-5-(3-trifluoromethylphenyl)-6-[4-(2,2-dimethyl-1,3-dioxolane)methoxy]pyrimidine* (**8f**). Method A. Light yellow solid 0.64 g, yield 68%; m.p. 126.0–126.8 °C; IR: υ_max_/cm^−1^ 3467 (NH), 3418 (NH), 3336 (NH), 1650 (C=N), 1625 (C=N), 1593 (C=C), 1556 (C=C), 1523 (C=C), 1163 (C-O-C), 1118 (C-O-C), 1106 (C-O-C), 1068 (C-O-C); ^1^H-NMR (CDCl_3_) δ 7.61 (s, 1H, Ar-*H*), 7.56–7.49 (m, 3H, Ar-*H*), 4.73 (s, 2H, N*H*_2_), 4.55 (s, 2H, N*H*_2_), 4.36 (dd, *J*_1_ = 10.6, *J*_2_ = 4.2, 1H, OC*H*_2_), 4.31–4.26 (m, 1H, C*H*), 4.22 (dd, *J*_1_ = 10.6, *J*_2_ = 5.8, 1H, OC*H*_2_), 3.96 (dd, *J*_1_ = 8.4, *J*_2_ = 6.4, 1H, OC*H*_2_), 3.70 (dd, *J*_1_ = 8.4, *J*_2_ = 6.0, 1H, OC*H*_2_), 1.32 (s, 3H, C*H*_3_), 1.25 (s, 3H, C*H*_3_); ES-MS 385.1 (M + H)^+^; HRMS Calcd. for C_17_H_20_F_3_N_4_O_3_^+^ 385.1488, found 385.1491.

*(R)-2,4-Diamino-5-(4-trifluoromethoxyphenyl)-6-[4-(2,2-dimethyl-1,3-dioxolane)methoxy]pyrimidine* (**8g**). Method A. Yellow solid 0.77 g, yield 70%; m.p. 37.1–38.0 °C; IR: υ_max_/cm^−1^ 3482 (NH), 3368 (NH), 1611 (C=N), 1560 (C=C), 1510 (C=C), 1374 (C-F), 1161 (C-O-C), 1092 (C-O-C), 1052 (C-O-C); ^1^H-NMR (CDCl_3_) δ 7.35 (d, *J* = 8.4, 2H, Ar-*H*), 7.24 (d, *J* = 8.4, 1H, Ar-*H*), 4.71 (s, 2H, N*H*_2_), 4.54 (s, 2H, N*H*_2_), 4.38 (dd, *J*_1_ = 10.8, *J*_2_ = 4.0, 1H, OC*H*_2_), 4.32–4.27 (m, 1H, C*H*), 4.21 (dd, *J*_1_ = 10.8, *J*_2_ = 5.6, 1H, OC*H*_2_*)*, 3.96 (dd, *J*_1_ = 8.4, *J*_2_ = 6.4, 1H, OC*H*_2_), 3.72 (dd, *J*_1_ = 8.4, *J*_2_ = 6.4, 1H, OC*H*_2_), 1.32 (s, 3H, C*H*_3_), 1.23 (s, 3H, C*H*_3_); ES-MS 401.1(M + H)^+^; HRMS Calcd. for C_17_H_20_F_3_N_4_O_4_^+^ 401.1437, found 401.1434.

*(R)-2,4-Diamino-5-(3-trifluoromethoxyphenyl)-6-[4-(2,2-dimethyl-1,3-dioxolane)methoxy]pyrimidine* (**8h**). Method A. Yellow solid 0.72 g, yield 66%; m.p. 36.2–37.1 °C; IR: υ_max_/cm^−1^ 3488 (NH), 3361 (NH), 1611 (C=N), 1560 (C=C), 1379 (C-F), 1158 (C-O-C), 1082 (C-O-C), 1047 (C-O-C); ^1^H-NMR (CDCl_3_) δ 7.41 (t, *J* = 8.0, 1H, Ar-*H*), 7.28–7.26 (m, 1H Ar-*H*), 7.21 (s, 1H, Ar-*H*), 7.14 (d, *J* = 8.4, 1H, Ar-*H*), 4.73 (s, 2H, N*H*_2_), 4.58 (s, 2H, N*H*_2_), 4.36 (dd, *J*_1_ = 10.8, *J*_2_ = 4.0, 1H, OC*H*_2_), 4.32–4.26 (m, 1H, C*H*), 4.21 (dd, *J*_1_ = 10.8, *J*_2_ = 6.0, 1H, OC*H*_2_), 3.97 (dd, *J*_1_ = 8.4, *J*_2_ = 6.4, 1H, OC*H*_2_), 3.72 (dd, *J*_1_ = 8.4, *J*_2_ = 6.0, 1H, OC*H*_2_), 1.32 (s, 3H, C*H*_3_), 1.27 (s, 3H, C*H*_3_); ES-MS 401.1 (M + H)^+^; HRMS Calcd. for C_17_H_20_F_3_N_4_O_4_^+^ 401.1437, found 401.1440.

*(R)-2,4-Diamino-5-(4-cyanophenyl)-6-[4-(2,2-dimethyl-1,3-dioxolane)methoxy]pyrimidine* (**8i**). Method A. White solid 0.62 g, yield 67%; m.p. 209.3–209.9 °C; IR: υ_max_/cm^−1^ 3482 (NH), 3456 (NH), 3370 (NH), 3332 (NH), 2228 (CN), 1655 (C=N), 1621 (C=N), 1585 (C=C), 1564 (C=C), 1157 (C-O-C), 1091 (C-O-C), 1051 (C-O-C); ^1^H-NMR (CDCl_3_) δ 7.68 (d, *J* = 8.4, 2H, Ar-*H*), 7.47 (d, *J* = 8.4, 2H Ar-*H*), 4.76 (s, 2H, N*H*_2_), 4.57 (s, 2H, N*H*_2_), 4.36 (dd, *J*_1_ = 10.8, *J*_2_ = 4.0, 1H, OC*H*_2_), 4.32–4.27 (m, 1H, C*H*), 4.23 (dd, *J*_1_ = 10.8, *J*_2_ = 6.0, 1H, OC*H*_2_*)*, 3.97 (dd, *J*_1_ = 8.4, *J*_2_ = 6.4, 1H, OC*H*_2_), 3.70 (dd, *J*_1_ = 8.4, *J*_2_ = 6.0, 1H, OC*H*_2_), 1.32 (s, 3H, C*H*_3_), 1.28 (s, 3H, C*H*_3_); ES-MS 342.1 (M + H)^+^; HRMS Calcd. for C_17_H_20_N_5_O_3_^+^ 342.1566, found 342.1570.

*(R)-2,4-Diamino-5-(4-methoxyphenyl)-6-[4-(2,2-dimethyl-1,3-dioxolane)methoxy]pyrimidine* (**8j**). Method A. White solid 0.64 g, yield 68%; m.p. 119.7–120.2 °C; IR: υ_max_/cm^−1^ 3456 (NH), 3348 (NH), 1643 (C=N), 1617(C=N), 1594 (C=C), 1560 (C=C), 1512 (C=C), 1171 (C-O-C), 1082 (C-O-C), 1050 (C-O-C); ^1^H-NMR (CDCl_3_) δ 7.21 (d, *J* = 8.8, 2H, Ar-*H*), 6.93 (d, *J* = 8.8, 2H, Ar-*H*), 4.65 (s, 2H, N*H*_2_), 4.53 (s, 2H, N*H*_2_), 4.37 (dd, *J*_1_ = 10.8, *J*_2_ = 4.0, 1H, OC*H*_2_), 4.32–4.26 (m, 1H, C*H*), 4.19 (dd, *J*_1_ = 10.8, *J*_2_ = 6.0, 1H, OC*H*_2_), 3.96 (dd, *J*_1_ = 8.4, *J*_2_ = 6.4, 1H, OC*H*_2_), 3.82 (s, 3H, OC*H*_3_), 3.74 (dd, *J*_1_ = 8.4, *J*_2_ = 6.4, 1H, OC*H*_2_), 1.32 (s, 3H, C*H*_3_), 1.28 (s, 3H, C*H*_3_); ES-MS 347.2 (M + H)^+^; HRMS Calcd. for C_17_H_23_N_4_O_4_^+^ 347.1719, found 347.1723.

*(R)-2,4-Diamino-5-(3,4,5-trimethoxyphenyl)-6-[4-(2,2-dimethyl-1,3-dioxolane)methoxy]pyrimidine* (**8k**). Method A. White solid 0.70 g, yield 63%; m.p. 183.8–184.1 °C; IR: υ_max_/cm^−1^ 3458 (NH), 3421 (NH), 3339 (NH), 1661 (C=N), 1631(C=N), 1588 (C=C), 1557 (C=C), 1511 (C=C), 1164 (C-O-C), 1128 (C-O-C), 1074 (C-O-C); ^1^H-NMR (CDCl_3_) δ 6.54 (s, 2H, Ar-*H*), 4.67 (s, 2H, N*H*_2_), 4.62 (s, 2H, N*H*_2_), 4.38–4.31 (m, 2H, OC*H*_2_), 4.27–4.20 (m, 1H, C*H*), 4.01 (dd, *J*_1_ = 8.4, *J*_2_ = 6.4, 1H, OC*H*_2_), 3.87 (s, 3H, OC*H*_3_), 3.84 (s, 6H, OC*H*_3_ × 2), 3.77 (dd, *J*_1_ = 8.0, *J*_2_ = 6.0, 1H, OC*H*_2_), 1.32 (s, 3H, C*H*_3_), 1.28 (s, 3H, C*H*_3_); ES-MS 407.2 (M + H)^+^; HRMS Calcd. for C_19_H_27_N_4_O_6_^+^ 407.1931, found 407.1927.

*(R)-2,4-Diamino-5-[3-(2,2,2-trifloroethoxymethyl)phenyl]-6-[4-(2,2-dimethyl-1,3-dioxolane)methoxy]-pyrimidine* (**8l**). Method A. Yellow solid 0.72 g, yield 62%; m.p. 32.2–33.2 °C; IR: υ_max_/cm^−1^ 3485 (NH), 3367 (NH), 1609 (C=N), 1559 (C=C), 1491 (C=C), 1373 (C-F), 1159 (C-O-C), 1093 (C-O-C), 1049 (C-O-C); ^1^H-NMR (CDCl_3_) δ 7.40 (t, *J* = 7.6, 1H, Ar-*H*), 7.30–7.27 (m, 3H, Ar-*H*), 4.68 (s, 4H, C*H*_2_, N*H*_2_), 4.56 (s, 2H, N*H*_2_), 4.35 (dd, *J*_1_ = 10.8, *J*_2_ = 4.0, 1H, OC*H*_2_), 4.32–4.26 (m, 1H, C*H*), 4.22 (dd, *J*_1_ = 10.4, *J*_2_ = 5.6, 1H, OC*H*_2_*)*, 3.96 (dd, *J*_1_ = 8.4, *J*_2_ = 6.4, 1H, OC*H*_2_), 3.87 (q, *J* = 8.8, 2H, CF_3_C*H*_2_), 3.73 (dd, *J*_1_ = 8.0, *J*_2_ = 5.8, 1H, OC*H*_2_), 1.31 (s, 3H, C*H*_3_), 1.25 (s, 3H, C*H*_3_); ES-MS 429.2(M + H)^+^; HRMS Calcd. for C_19_H_24_F_3_N_4_O_4_^+^ 429.1750, found 429.1747.

*(R)-2,4-Diamino-5-[3,5-dimethyl-4-(N-methoxyaminosulfonyl)phenyl]-6-[4-(2,2-dimethyl-1,3-dioxolane)-methoxy]pyrimidine* (**8m**). Method A. Light yellow solid 0.69 g, yield 56%; m.p. 157.7–158.8 °C; IR: υ_max_/cm^−1^ 3485 (NH), 3446 (NH), 3347 (NH), 3223 (NH), 1640 (C=N), 1617 (C=N), 1591 (C=C), 1562 (C=C), 1490 (C=C), 1170 (C-O-C), 1096 (C-O-C), 1053 (C-O-C); ^1^H-NMR (CDCl_3_) δ 7.43 (s, 1H, N*H*), 7.18 (s, 2H, Ar-*H*), 4.74 (s, 2H, N*H*_2_), 4.61 (s, 2H, N*H*_2_), 4.35 (dd, *J*_1_ = 10.2, *J*_2_ = 5.0, 1H, OC*H*_2_), 4.32–4.27 (m, 1H, C*H*), 4.24 (dd, *J*_1_ = 10.2, *J*_2_ = 5.0, 1H, OC*H*_2_), 3.99 (dd, *J*_1_ = 8.2, *J*_2_ = 6.2, 1H, OC*H*_2_), 3.75–3.72 (m, 4H, OC*H*_3_ and OC*H*_2_), 2.69 (s, 6H, C*H*_3_ × 2), 1.33 (s, 3H, C*H*_3_), 1.29 (s, 3H, C*H*_3_); ES-MS 454.2 (M + H)^+^; HRMS Calcd. for C_19_H_28_N_5_O_6_S^+^ 454.1760, found 454.1756.

*(R)-2,4-Diamino-5-[4-(morpholine-4-carbonyl)phenyl]-6-[4-(2,2-dimethyl-1,3-dioxolane)methoxy]pyrimidine* (**8n**). Method A. Yellow solid 0.90 g, yield 77%; m.p. 189.4–190.5 °C; IR: υ_max_/cm^−1^ 3492 (NH), 3430 (NH), 3334 (NH), 1659 (C=N), 1611(C=N), 1588 (C=C), 1560 (C=C), 1516 (C=C), 1488 (C=C), 1156 (C-O-C), 1114 (C-O-C), 1070 (C-O-C), 1051(C-O-C); ^1^H-NMR (CDCl_3_) δ 7.44 (d, *J* = 8.4, 2H, Ar-*H*), 7.38 (d, *J* = 8.4, 2H, Ar-*H*), 4.71 (s, 2H, N*H*_2_), 4.57 (s, 2H, N*H*_2_), 4.35 (dd, *J*_1_ = 10.8, *J*_2_ = 4.2, 1H, OC*H*_2_), 4.32–4.29 (m, 1H, C*H*), 4.22 (dd, *J*_1_ = 10.8, *J*_2_ = 5.6, 1H, OC*H*_2_), 3.97 (dd, *J*_1_ = 8.4, *J*_2_ = 6.4, 1H, OC*H*_2_), 3.82–3.51 (m, 8H, C*H*_2_ × 4), 3.73 (dd, *J*_1_ = 8.0, *J*_2_ = 6.0, 1H, OC*H*_2_), 1.32 (s, 3H, C*H*_3_), 1.27 (s, 3H, C*H*_3_); ES-MS 430.2 (M + H)^+^; HRMS Calcd. for C_21_H_28_N_5_O_5_^+^ 430.2090, found 430.2092.

*(R)-2,4-Diamino-5-[4-(3-trifloromethoxyanilinocarbonyl)phenyl]-6-[4-(2,2-dimethyl-1,3-dioxolane)-methoxy]pyrimidine* (**8o**). Method A. White solid 0.73 g, yield 52%; m.p. 122.9–124.5 °C; IR: υ_max_/cm^−1^ 3496 (NH), 3340 (NH), 3192 (CONH), 1655 (C=N), 1607 (C=C), 1565 (C=C), 1492 (C=C), 1156 (C-O-C), 1082 (C-O-C); ^1^H-NMR (CDCl_3_) δ 8.07 (s, 1H, N*H*), 7.85 (d, *J* = 8.4, 2H, Ar-H), 7.71 (s, 1H, Ar-*H*), 7.54 (dd, *J*_1_ = 8.0, *J*_2_ = 1.2, 1H, Ar-*H*), 7.45 (d, *J* = 8.4, 2H, Ar-*H*), 7.38 (t, *J* = 8.0, 1H, Ar-*H*), 7.02 (d, *J* = 8.0, 1H, Ar-*H*), 4.74 (s, 2H, N*H*_2_), 4.58 (s, 2H, N*H*_2_), 4.35 (dd, *J*_1_ = 9.6, *J*_2_ = 3.8, 1H, OC*H*_2_), 4.32–4.29 (m, 1H, C*H*), 4.27 (dd, *J*_1_ = 9.6, *J*_2_ = 4.6, 1H, OC*H*_2_), 3.99 (dd, *J*_1_ = 8.4, *J*_2_ = 6.0, 1H, OC*H*_2_), 3.73 (dd, *J*_1_ = 8.2, *J*_2_ = 5.4, 1H, OC*H*_2_), 1.30 (s, 3H, C*H*_3_), 1.29 (s, 3H, C*H*_3_); ES-MS 520.2 (M + H)^+^; HRMS Calcd. for C_24_H_25_F_3_N_5_O_5_^+^ 520.1808, found 520.1810.

*(R)-2,4-Diamino-5-(4-methoxycarbonylphenyl)-6-[4-(2,2-dimethyl-1,3-dioxolane)methoxy]pyrimidine* (**8p**). Method B. White solid 0.64 g, yield 63%; m.p. 151.3–152.4 °C; IR: υ_max_/cm^−1^ 3500 (NH), 3443 (NH), 3391 (NH), 3335 (NH), 1709 (C=O), 1657 (C=N), 1608 (C=N), 1568 (C=C), 1556 (C=C), 1513 (C=C), 1194 (C-O-C), 1180 (C-O-C), 1152 (C-O-C), 1116 (C-O-C), 1067 (C-O-C); ^1^H-NMR (CDCl_3_) δ 8.05 (dd, *J*_1_ = 6.8, *J*_2_ = 1.6, 2H, Ar-*H*), 7.42 (dd, *J*_1_ = 6.8, *J*_2_ = 1.6, 2H, Ar-*H*), 4.72 (s, 2H, N*H*_2_), 4.58 (s, 2H, N*H*_2_), 4.36 (dd, *J*_1_ = 10.4, *J*_2_ = 4.0, 1H, OC*H*_2_), 4.31–4.26 (m, 1H, C*H*), 4.22 (dd, *J*_1_ = 10.4, *J*_2_ = 6.0, 1H, OC*H*_2_), 3.96 (dd, *J*_1_ = 8.0, *J*_2_ = 6.0, 1H, OC*H*_2_), 3.93 (s, 3H, OC*H*_3_), 3.72 (dd, *J*_1_ = 8.4, *J*_2_ = 6.0, 1H, OC*H*_2_), 1.32 (s, 3H, C*H*_3_), 1.27 (s, 3H, C*H*_3_); ES-MS 375.2(M + H)^+^; HRMS Calcd. for C_18_H_23_N_4_O_5_^+^ 375.1668, found 375.1664.

*(R)-2,4-Diamino-5-(3-methoxycarbonylphenyl)-6-[4-(2,2-dimethyl-1,3-dioxolane)methoxy]pyrimidine* (**8q**). Method B. White solid 0.66 g, yield 65%; m.p. 181.4–181.6 °C; IR: υ_max_/cm^−1^ 3477 (NH), 3373 (NH), 3326 (NH), 1708 (C=O), 1651 (C=N), 1626 (C=N), 1597 (C=C), 1558 (C=C), 1490 (C=C), 1158 (C-O-C), 1115 (C-O-C), 1084 (C-O-C), 1049 (C-O-C); ^1^H-NMR (CDCl_3_) δ 8.00 (t, *J* = 1.6, 1H, Ar-*H*), 7.96 (dt, *J*_1_ = 7.6, *J*_2_ = 1.6, 1H, Ar-*H*), 7.52 (dt, *J*_1_ = 7.6, *J*_2_ = 1.6, 1H, Ar-*H*), 7.47 (t, *J* = 7.6, 1H, Ar-*H*), 4.71 (s, 2H, N*H*_2_), 4.54 (s, 2H, N*H*_2_), 4.37 (dd, *J*_1_ = 10.8, *J*_2_ = 4.0, 1H, OC*H*_2_), 4.30–4.25 (m, 1H, C*H*), 4.19 (dd, *J*_1_ = 10.8, *J*_2_ = 6.0, 1H, OC*H*_2_), 3.96 (dd, *J*_1_ = 8.4, *J*_2_ = 6.0, 1H, OC*H*_2_), 3.92 (s, 3H, OC*H*_3_), 3.73 (dd, *J*_1_ = 8.4, *J*_2_ = 6.0, 1H, OC*H*_2_), 1.31 (s, 3H, C*H*_3_), 1.25 (s, 3H, C*H*_3_); ES-MS 375.2(M + H)^+^; HRMS Calcd. for C_18_H_23_N_4_O_5_^+^ 375.1668, found 375.1670.

*(S)-2,4-Diamino-5-(4-chlorophenyl)-6-[4-(2,2-dimethyl-1,3-dioxolane)methoxy]pyrimidine* (**9a**). Method A. White solid 0.58 g, yield 61%; m.p. 171.9–172.1 °C; IR: υ_max_/cm^−1^ 3475 (NH), 3454 (NH), 3396 (NH), 3315 (NH), 1649 (C=N), 1620 (C=N), 1593 (C=C), 1549 (C=C), 1479 (C=C), 1160 (C-O-C), 1141 (C-O-C), 1089(C-O-C), 794 (C-Cl); ^1^H-NMR (CDCl_3_) δ 7.36 (d, *J* = 8.2, 2H, Ar-*H*), 7.25 (d, *J* = 8.2, 2H, Ar-*H*), 4.69 (s, 2H, N*H*_2_), 4.53 (s, 2H, N*H*_2_), 4.36 (dd, *J*_1_ = 10.8, *J*_2_ = 4.0, 1H, OC*H*_2_), 4.31–4.26 (m, 1H, C*H*), 4.20 (dd, *J*_1_ = 10.8, *J*_2_ = 5.6, 1H, OC*H*_2_), 3.97 (dd, *J*_1_ = 8.4, *J*_2_ = 6.4, 1H, OC*H*_2_), 3.72 (dd, *J*_1_ = 8.4, *J*_2_ = 6.0, 1H, OC*H*_2_), 1.32 (s, 3H, C*H*_3_), 1.28 (s, 3H, C*H*_3_); ES-MS 351.1 (M + H)^+^; HRMS Calcd. for C_16_H_20_ClN_4_O_3_^+^ 351.1224, found 351.1220.

*(S)-2,4-Diamino-5-(4-fluorophenyl)-6-[4-(2,2-dimethyl-1,3-dioxolane)methoxy]pyrimidine* (**9b**). Method A. White solid 0.57 g, yield 62%; m.p. 164.1–164.5 °C; IR: υ_max_/cm^−1^ 3527 (NH), 3482 (NH), 3370 (NH), 3312 (NH), 1646 (C=N), 1626 (C=N), 1593 (C=C), 1558 (C=C), 1512 (C=C), 1484 (C=C), 1380 (C-F), 1159 (C-O-C), 1130 (C-O-C), 1091 (C-O-C); ^1^H-NMR (CDCl_3_) δ 7.26 (dd, *J*_1_ = 8.8, *J*_2_ = 5.2, 2H, Ar-*H*), 7.08 (t, *J* = 8.8, 2H, Ar-*H*), 4.67 (s, 2H, N*H*_2_), 4.50 (s, 2H, N*H*_2_), 4.36 (dd, *J*_1_ = 10.8, *J*_2_ = 4.0, 1H, OC*H*_2_), 4.31–4.26 (m, 1H, C*H*), 4.20 (dd, *J*_1_ = 10.8, *J*_2_ = 5.6, 1H, OC*H*_2_), 3.96 (dd, *J*_1_ = 8.4, *J*_2_ = 6.4, 1H, OC*H*_2_), 3.72 (dd, *J*_1_ = 8.4, *J*_2_ = 6.0, 1H, OC*H*_2_), 1.32 (s, 3H, C*H*_3_), 1.27 (s, 3H, C*H*_3_); ES-MS 335.2 (M + H)^+^; HRMS Calcd. for C_16_H_20_FN_4_O_3_^+^ 335.1519, found 335.1521.

*(S)-2,4-Diamino-5-(3,4-dichlorophenyl)-6-[4-(2,2-dimethyl-1,3-dioxolane)methoxy]pyrimidine* (**9c**). Method A. White solid 0.58 g, yield 55%; m.p. 167.3–167.7 °C; IR: υ_max_/cm^−1^ 3487 (NH), 3434(NH), 3378 (NH), 3318 (NH), 1650 (C=N), 1624 (C=N), 1600 (C=C), 1558 (C=C), 1487 (C=C), 1155 (C-O-C), 1093 (C-O-C), 1047 (C-O-C), 795 (C-Cl); ^1^H-NMR (CDCl_3_) δ 7.47 (d, *J* = 8.4, 1H, Ar-*H*), 7.44 (d, *J* = 2.0, 1H, Ar-*H*), 7.18 (dd, *J*_1_ = 8.4, *J*_2_ = 2.0, 1H, Ar-*H*), 4.71 (s, 2H, N*H*_2_), 4.54 (s, 2H, N*H*_2_)**,** 4.35 (dd, *J*_1_ = 10.4, *J*_2_ = 4.4, 1H, OC*H*_2_), 4.33–4.27 (m, 1H, C*H*), 4.22 (dd, *J*_1_ = 10.4, *J*_2_ = 5.2, 1H, OC*H*_2_), 3.99 (dd, *J*_1_ = 8.4, *J*_2_ = 6.0, 1H, OC*H*_2_), 3.73 (dd, *J*_1_ = 8.4, *J*_2_ = 6.0, 1H, OC*H*_2_), 1.34 (s, 3H, C*H*_3_), 1.30 (s, 3H, C*H*_3_); ES-MS 385.1 (M + H)^+^; HRMS Calcd. for C_16_H_19_Cl_2_N_4_O_3_^+^ 385.0834, found 385.0838.

*(S)-2,4-Diamino-5-(3-chloro-4-fluorophenyl)-6-[4-(2,2-dimethyl-1,3-dioxolane)methoxy]pyrimidine* (**9d**). Method A. White solid 0.56 g, yield 56%; m.p. 152.4–152.6 °C; IR: υ_max_/cm^−1^ 3527 (NH), 3477 (NH), 3377 (NH), 3355 (NH), 1626 (C=N), 1598 (C=C), 1559 (C=C), 1482 (C=C), 1376 (C-F), 1163 (C-O-C), 1128 (C-O-C), 1093 (C-O-C), 1043 (C-O-C), 798 (C-Cl); ^1^H-NMR (CDCl_3_) δ 7.37 (dd, *J*_1_ = 7.0, *J*_2_ = 1.8, 1H, Ar-*H*), 7.21–7.14 (m, 2H, Ar-*H*), 4.71 (s, 2H, N*H*_2_), 4.53 (s, 2H, N*H*_2_), 4.35 (dd, *J*_1_ = 10.6, *J*_2_ = 4.2, 1H, OC*H*_2_), 4.32–4.27 (m, 1H, C*H*), 4.22 (dd, *J*_1_ = 10.6, *J*_2_ = 5.4, 1H, OC*H*_2_), 3.98 (dd, *J*_1_ = 8.4, *J*_2_ = 6.4, 1H, OC*H*_2_), 3.72 (dd, *J*_1_ = 8.4, *J*_2_ = 6.0, 1H, OC*H*_2_), 1.33 (s, 3H, C*H*_3_), 1.29 (s, 3H, C*H*_3_); ES-MS 369.1 (M + H)^+^; HRMS Calcd. for C_16_H_19_ClFN_4_O_3_^+^, 369.1130, found 369.1132.

*(S)-2,4-Diamino-5-(4-trifluoromethylphenyl)-6-[4-(2,2-dimethyl-1,3-dioxolane)methoxy]pyrimidine* (**9e**). Method A. Light yellow solid 0.64 g, yield 61%; m.p. 130.0–131.6 °C; IR: υ_max_/cm^−1^ 3467 (NH), 3418 (NH), 3336 (NH), 1650 (C=N), 1625 (C=N), 1593 (C=C), 1556 (C=C), 1523 (C=C), 1163 (C-O-C), 1118 (C-O-C), 1106 (C-O-C), 1068 (C-O-C); ^1^H-NMR (CDCl_3_) δ 7.64 (d, *J* = 8.0, 2H, Ar-*H*), 7.46 (d, *J* = 8.0, 2H, Ar-*H*), 4.72 (s, 2H, N*H*_2_), 4.55 (s, 2H, N*H*_2_), 4.37 (dd, *J*_1_ = 10.8, *J*_2_ = 4.2, 1H, OC*H*_2_), 4.32–4.27 (m, 1H, C*H*), 4.22 (dd, *J*_1_ = 10.8, *J*_2_ = 5.6, 1H, OC*H*_2_), 3.97 (dd, *J*_1_ = 8.4, *J*_2_ = 6.0, 1H, OC*H*_2_), 3.71 (dd, *J*_1_ = 8.4, *J*_2_ = 6.0, 1H, OC*H*_2_), 1.32 (s, 3H, C*H*_3_), 1.24 (s, 3H, C*H*_3_); ES-MS 385.1 (M + H)^+^; HRMS Calcd. for C_17_H_20_F_3_N_4_O_3_^+^ 385.1488, found 385.1490.

*(S)-2,4-Diamino-5-(3-trifluoromethylphenyl)-6-[4-(2,2-dimethyl-1,3-dioxolane)methoxy]pyrimidine* (**9f**). Method A. Light yellow solid 0.74 g, yield 71%; m.p. 126.3–126.5 °C; IR: υ_max_/cm^−1^ 3512 (NH), 3420 (NH), 3382 (NH), 3327 (NH), 1650 (C=N), 1612 (C=N), 1593 (C=C), 1559 (C=C), 1171 (C-O-C), 1128 (C-O-C), 1094 (C-O-C), 1072 (C-O-C), 1046 (C-O-C); ^1^H-NMR (CDCl_3_) δ 7.61 (s, 1H, Ar-*H*), 7.56–7.49 (m, 3H, Ar-*H*), 4.73 (s, 2H, N*H*_2_), 4.55 (s, 2H, N*H*_2_), 4.36 (dd, *J*_1_ = 10.8, *J*_2_ = 4.0, 1H, OC*H*_2_), 4.31–4.25 (m, 1H, C*H*), 4.21 (dd, *J*_1_ = 10.8, *J*_2_ = 5.6, 1H, OC*H*_2_), 3.97 (dd, *J*_1_ = 8.2, *J*_2_ = 6.0, 1H, OC*H*_2_), 3.71 (dd, *J*_1_ = 8.2, *J*_2_= 6.0, 1H, OC*H*_2_), 1.32 (s, 3H, C*H*_3_), 1.25 (s, 3H, C*H*_3_); ES-MS 385.1 (M + H)^+^; HRMS Calcd. for C_17_H_20_F_3_N_4_O_3_^+^ 385.1488, found 385.1491.

*(S)-2,4-Diamino-5-(4-trifluoromethoxyphenyl)-6-[4-(2,2-dimethyl-1,3-dioxolane)methoxy]pyrimidine* (**9g**). Method A. Yellow solid 0.80 g, yield 73%; m.p. 36.7–37.7 °C; IR: υ_max_/cm^−1^ 3487 (NH), 3358 (NH), 1612 (C=N), 1559 (C=C), 1510 (C=C), 1374 (C-F), 1160 (C-O-C), 1092 (C-O-C), 1053 (C-O-C); ^1^H-NMR (CDCl_3_) δ 7.35 (d, *J* = 8.4, 2H, Ar-*H*), 7.24 (d, *J* = 8.4, 1H, Ar-*H*), 4.70 (s, 2H, N*H*_2_), 4.53 (s, 2H, N*H*_2_), 4.38 (dd, *J*_1_ = 11.2, *J*_2_ = 4.0, 1H, OC*H*_2_), 4.32–4.26 (m, 1H, C*H*), 4.21 (dd, *J*_1_ = 11.2, *J*_2_ = 5.4, 1H, OC*H*_2_*)*, 3.96 (dd, *J*_1_ = 8.4, *J*_2_ = 6.4, 1H, OC*H*_2_), 3.72 (dd, *J*_1_ = 8.2, *J*_2_ = 6.2, 1H, OC*H*_2_), 1.32 (s, 3H, C*H*_3_), 1.24 (s, 3H, C*H*_3_); ES-MS 401.1(M + H)^+^; HRMS Calcd. for C_17_H_20_F_3_N_4_O_4_^+^ 401.1437, found 401.1440.

*(S)-2,4-Diamino-5-(3-trifluoromethoxyphenyl)-6-[4-(2,2-dimethyl-1,3-dioxolane)methoxy]pyrimidine* (**9h**). Method A. Yellow solid 0.73 g, yield 67%; m.p. 33.7–34.7 °C; IR: υ_max_/cm^−1^ 3490 (NH), 3360 (NH), 1611 (C=N), 1561 (C=C), 1379 (C-F), 1158 (C-O-C), 1082 (C-O-C), 1047 (C-O-C); ^1^H-NMR (CDCl_3_) δ 7.41 (t, *J* = 8.0, 1H, Ar-*H*), 7.28–7.26 (m, 1H Ar-*H*), 7.21 (s, 1H, Ar-*H*) 7.14 (dt, *J*_1_ = 8.4, *J*_2_ = 1.2, 1H, Ar-*H*), 4.75 (s, 2H, N*H*_2_), 4.60 (s, 2H, N*H*_2_), 4.36 (dd, *J*_1_ = 10.8, *J*_2_ = 4.2, 1H, OC*H*_2_), 4.32–4.26 (m, 1H, C*H*), 4.21 (dd, *J*_1_ = 10.8, *J*_2_ = 5.6, 1H, OC*H*_2_*)*, 3.97 (dd, *J*_1_ = 8.4, *J*_2_ = 6.4, 1H, OC*H*_2_), 3.72 (dd, *J*_1_ = 8.4, *J*_2_ = 6.0, 1H, OC*H*_2_), 1.32 (s, 3H, C*H*_3_), 1.27 (s, 3H, C*H*_3_); ES-MS 401.1(M + H)^+^; HRMS Calcd. for C_17_H_20_F_3_N_4_O_4_^+^ 401.1437, found 401.1435.

*(S)-2,4-Diamino-5-(4-cyanophenyl)-6-[4-(2,2-dimethyl-1,3-dioxolane)methoxy]pyrimidine* (**9i**). Method A. White solid 0.66 g, yield 71%; m.p. 221.4–221.9 °C; IR: υ_max_/cm^−1^ 3482 (NH), 3456 (NH), 3371 (NH), 3332 (NH), 2228 (CN), 1654 (C=N), 1620 (C=N), 1586 (C=C), 1564 (C=C), 1157 (C-O-C), 1091 (C-O-C), 1051 (C-O-C); ^1^H-NMR (CDCl_3_) δ 7.67 (d, *J* = 8.4, 2H, Ar-*H*), 7.47 (d, *J* = 8.4, 2H Ar-*H*), 4.75 (s, 2H, N*H*_2_), 4.57 (s, 2H, N*H*_2_), 4.36 (dd, *J*_1_ = 10.8, *J*_2_ = 4.0, 1H, OC*H*_2_), 4.32–4.27 (m, 1H, C*H*), 4.23 (dd, *J*_1_ = 10.4, *J*_2_ = 4.2, 1H, OC*H*_2_), 3.97 (dd, *J*_1_ = 8.4, *J*_2_ = 6.4, 1H, OC*H*_2_), 3.70 (dd, *J*_1_ = 8.4, *J*_2_ = 6.0, 1H, OC*H*_2_), 1.32 (s, 3H, C*H*_3_), 1.28 (s, 3H, C*H*_3_); ES-MS 342.1 (M + H)^+^; HRMS Calcd. for C_17_H_20_N_5_O_3_^+^ 342.1566, found 342.1564.

*(S)-2,4-Diamino-5-(4-methoxyphenyl)-6-[4-(2,2-dimethyl-1,3-dioxolane)methoxy]pyrimidine* (**9j**). Method A. White solid 0.57 g, yield 60%; m.p. 120.1–121.3 °C; IR: υ_max_/cm^−1^ 3458 (NH), 3349 (NH), 1620 (C=N), 1594 (C=C), 1558 (C=C), 1513 (C=C), 1157 (C-O-C), 1085 (C-O-C), 1046 (C-O-C); ^1^H-NMR (CDCl_3_) δ 7.21 (d, *J* = 8.8, 2H, Ar-*H*), 6.93 (d, *J* = 8.8, 2H, Ar-*H*), 4.65 (s, 2H, N*H*_2_), 4.53 (s, 2H, N*H*_2_)，4.37 (dd, *J*_1_ = 10.8，*J*_2_ = 4.0, 1H, OC*H*_2_), 4.32–4.26 (m, 1H, C*H*), 4.19 (dd, *J*_1_ = 10.8, *J*_2_ = 6.0, 1H, OC*H*_2_), 3.96 (dd, *J*_1_ = 8.4, *J*_2_ = 6.0, 1H, OC*H*_2_), 3.82 (s, 3H, OC*H*_3_), 3.74 (dd, *J*_1_ = 8.4, *J*_2_ = 6.0, 1H, OC*H*_2_), 1.32 (s, 3H, C*H*_3_), 1.28 (s, 3H, C*H*_3_); ES-MS 347.2 (M + H)^+^; HRMS Calcd. for C_17_H_23_N_4_O_4_^+^ 347.1719, found 347.1721.

*(S)-2,4-Diamino-5-(3,4,5-trimethoxyphenyl)-6-[4-(2,2-dimethyl-1,3-dioxolane)methoxy]pyrimidine* (**9k**). Method A. White solid 0.61 g, yield 55%; m.p. 181.7–183.0 °C; IR: υ_max_/cm^−1^ 3458 (NH), 3421 (NH), 3339 (NH), 1660 (C=N), 1631 (C=N), 1586 (C=C), 1557 (C=C), 1510 (C=C), 1163 (C-O-C), 1129 (C-O-), 1075 (C-O-C); ^1^H-NMR (CDCl_3_) δ 6.55 (s, 2H, Ar-*H*), 4.66 (s, 2H, N*H*_2_), 4.61 (s, 2H, N*H*_2_), 4.38–4.31 (m, 2H, OC*H*_2_), 4.2–4.21 (m, 1H, C*H*), 4.00 (dd, *J*_1_ = 8.4, *J*_2_ = 6.4, 1H, OC*H*_2_), 3.87 (s, 3H, OC*H*_3_), 3.84 (s, 6H, OC*H*_3_ × 2), 3.77 (dd, *J*_1_ = 8.0, *J*_2_ = 6.0, 1H, OC*H*_2_), 1.32 (s, 3H, C*H*_3_), 1.28 (s, 3H, C*H*_3_); ES-MS 407.2 (M + H)^+^; HRMS Calcd. for C_19_H_27_N_4_O_6_^+^ 407.1931, found 407.1933.

*(S)-2,4-Diamino-5-[3-(2,2,2-trifluoroethoxymethyl)phenyl]-6-[4-(2,2-dimethyl-1,3-dioxolane)methoxy]-pyrimidine* (**9l**). Method A. Yellow solid 0.73 g, yield 62%; m.p. 32.8–33.7 °C; IR: υ_max_/cm^−1^ 3482 (NH), 3372 (NH), 1611 (C=N), 1559 (C=C), 1377 (C-F), 1159 (C-O-C), 1121 (C-O-C), 1093 (C-O-C), 1049 (C-O-C); ^1^H-NMR (CDCl_3_) δ 7.40 (t, *J* = 7.6, 1H, Ar-*H*), 7.30–7.27 (m, 3H, Ar-*H*), 4.68 (s, 4H, OC*H2* and N*H*_2_), 4.55 (s, 2H, N*H*_2_), 4.35 (dd, *J*_1_ = 10.4, *J*_2_ = 4.0, 1H, OC*H*_2_), 4.31–4.26 (m, 1H, C*H*), 4.22 (dd, *J*_1_ = 10.8, *J*_2_ = 5.6, 1H, OC*H*_2_*)*, 3.96 (dd, *J*_1_ = 8.4, *J*_2_ = 6.0, 1H, OC*H*_2_), 3.86 (q, *J* = 8.8, 2H, CF_3_C*H*_2_), 3.73 (dd, *J*_1_ = 8.4, *J*_2_ = 6.0, 1H, OC*H*_2_), 1.31 (s, 3H, C*H*_3_), 1.25 (s, 3H, C*H*_3_); ES-MS 429.2(M + H)^+^; HRMS Calcd. for C_19_H_24_F_3_N_4_O_4_^+^ 429.1750, found 429.1752.

*(S)-2,4-Diamino-5-[3,5-dimethyl-4-(N-methoxyaminosulfonyl)phenyl]-6-[4-(2,2-dimethyl-1,3-dioxolane)-methoxy]pyrimidine* (**9m**). Method A. Light yellow solid 0.75 g, yield 61%; m.p. 160.5–160.8 °C; IR: υ_max_/cm^−1^ 3484 (NH), 3446 (NH), 3347 (NH), 3223 (SO_2_NH), 1641 (C=N), 1619 (C=N), 1591 (C=C), 1562 (C=C), 1492 (C=C), 1173 (C-O-C), 1149 (C-O-C), 1099 (C-O-C), 1054 (C-O-C); ^1^H-NMR (CDCl_3_) δ 7.42 (s, 1H, N*H*), 7.18 (s, 2H, Ar-*H*), 4.73 (s, 2H, N*H*_2_), 4.60 (s, 2H, N*H*_2_), 4.35 (dd, *J*_1_ = 10.4, *J*_2_ = 4.0, 1H, OC*H*_2_), 4.32–4.27 (m, 1H, C*H*), 4.24 (dd, *J*_1_ = 10.4, *J*_2_ = 4.8, 1H, OC*H*_2_*)*, 3.99 (dd, *J*_1_ = 8.2, *J*_2_ = 6.2, 1H, OC*H*_2_), 3.75–3.72 (m, 4H, OC*H*_3_ and OC*H*_2_), 2.69 (s, 6H, C*H*_3_ × 2), 1.33 (s, 3H, C*H*_3_), 1.29 (s, 3H, C*H*_3_); ES-MS 454.2 (M + H)^+^; HRMS Calcd. for C_19_H_28_N_5_O_6_S^+^ 454.1760, found 454.1762.

*(S)-2,4-Diamino-5-[4-(morpholino-4-carbonyl)phenyl]-6-[4-(2,2-dimethyl-1,3-dioxolane)methoxy]pyrimidine* (**9n**). Method A. White solid 0.64 g, yield 55%; m.p. 186.1–187.2 °C; IR: υ_max_/cm^−1^ 3492 (NH), 3430 (NH), 3332 (NH), 1659 (C=N), 1610 (C=N), 1588 (C=C), 1558 (C=C), 1516 (C=C), 1488 (C=C), 1156 (C-O-C), 1114 (C-O-C), 1070 (C-O-C), 1050 (C-O-C); ^1^H-NMR (CDCl_3_) δ 7.45 (d, *J* = 8.4, 2H, Ar-*H*), 7.38 (d, *J* = 8.4, 2H, Ar-*H*), 4.82 (s, 2H, N*H*_2_), 4.67 (s, 2H, N*H*_2_), 4.38 (dd, *J*_1_ = 10.8, *J*_2_ = 4.4, 1H, OC*H*_2_), 4.32–4.28 (m, 1H, C*H*), 4.23 (dd, *J*_1_ = 10.8, *J*_2_ = 5.6, 1H, OC*H*_2_), 3.97 (dd, *J*_1_ = 8.2, *J*_2_ = 6.2, 1H, OC*H*_2_), 3.82–3.51 (m, 8H, C*H*_2_ × 4), 3.72 (dd, *J*_1_ = 8.4, *J*_2_ = 6.0, 1H, OC*H*_2_), 1.32 (s, 3H, C*H*_3_), 1.28 (s, 3H, C*H*_3_); ES-MS 430.2 (M + H)^+^; HRMS Calcd. for C_21_H_28_N_5_O_5_^+^ 430.2090, found 430.2088.

*(S)-2,4-Diamino-5-[4-(3-trifluoromethoxyanilinocarbonyl)phenyl]-6-[4-(2,2-dimethyl-1,3-dioxolane)-methoxy]pyrimidine* (**9o**). Method A. White solid 0.74 g, yield 52%; m.p. 83.7–85 °C; IR: υ_max_/cm^−1^ 3488 (NH), 3351 (NH), 3202 (CONH), 1657 (C=N), 1606 (C=C), 1565 (C=C), 1548 (C=C), 1492 (C=C), 1156 (C-O-C), 1082 (C-O-C); ^1^H-NMR (CDCl_3_) δ 8.04 (s, 1H, N*H*), 7.85 (d, *J* = 8.4, 2H, Ar-*H*), 7.71 (s, 1H, Ar-*H*), 7.54 (dd, *J*_1_ = 8.0, *J*_2_ = 1.2, 1H, Ar-H), 7.45 (d, *J* = 8.4, 2H, Ar-H), 7.39 (t, *J* = 8.0, 1H, Ar-*H*), 7.02 (dt, *J*_1_ = 8.0, *J*_2_ = 1.0, 1H, Ar-*H*), 4.74 (s, 2H, N*H*_2_), 4.59 (s, 2H, N*H*_2_), 4.35 (dd, *J*_1_ = 10.0, *J*_2_ = 4.0, 1H, OC*H*_2_), 4.32–4.29 (m, 1H, C*H*), 4.26 (dd, *J*_1_ = 10.0, *J*_2_ = 4.8, 1H, OC*H*_2_*)*, 3.99 (dd, *J*_1_ = 8.4, *J*_2_ = 6.0, 1H, OC*H*_2_), 3.73 (dd, *J*_1_ = 8.2, *J*_2_ = 5.4, 1H, OC*H*_2_), 1.30 (s, 3H, C*H*_3_), 1.29 (s, 3H, C*H*_3_); ES-MS 520.2 (M + H)^+^; HRMS Calcd. for C_24_H_25_F_3_N_5_O_5_^+^ 520.1808, found 520.1811.

*(S)-2,4-Diamino-5-(4-methoxycarbonylphenyl)-6-[4-(2,2-dimethyl-1,3-dioxolane)methoxy]pyrimidine* (**9p**). Method B. White solid 0.70 g, yield 69%; m.p. 146.3–147.4 °C; IR: υ_max_/cm^−1^ 3501 (NH), 3442 (NH), 3392 (NH), 3336 (NH), 1708 (C=O), 1658 (C=N), 1610 (C=N), 1555 (C=C), 1515 (C=C), 1153 (C-O-C), 1115 (C-O-C), 1067 (C-O-C); ^1^H-NMR (CDCl_3_) δ 8.05 (d, *J* = 8.4, 2H, Ar-*H*), 7.29 (d, *J* = 8.4, 2H, Ar-*H*), 4.72 (s, 2H, N*H*_2_), 4.58 (s, 2H, N*H*_2_), 4.36 (dd, *J*_1_ = 10.6, *J*_2_ = 4.2, 1H, OC*H*_2_), 4.31–4.26 (m, 1H, C*H*), 4.22 (dd, *J*_1_ = 10.6, *J*_2_ = 5.8, 1H, OC*H*_2_), 3.96 (dd, *J*_1_ = 8.4, *J*_2_ = 6.0, 1H, OC*H*_2_), 3.93 (s, 3H, OC*H*_3_), 3.71 (dd, *J*_1_ = 8.0, *J*_2_ = 6.0, 1H, OC*H*_2_), 1.32 (s, 3H, C*H*_3_), 1.27 (s, 3H, C*H*_3_); ES-MS 375.2(M + H)^+^; HRMS Calcd. for C_18_H_23_N_4_O_5_^+^ 375.1668, found 375.1670.

*(S)-2,4-Diamino-5-(3-methoxycarbonylphenyl)-6-[4-(2,2-dimethyl-1,3-dioxolane)methoxy]pyrimidine* (**9q**). Method B. White solid 0.80 g, yield 78%; m.p. 179.2–180.3 °C; IR: υ_max_/cm^−1^ 3480 (NH), 3371 (NH), 3326 (NH), 1708 (C=O), 1652 (C=N), 1626 (C=N), 1601 (C=C), 1592 (C=C), 1557 (C=C), 1158 (C-O-C), 1115 (C-O-C), 1084 (C-O-C), 1049 (C-O-C); ^1^H-NMR (CDCl_3_) δ 8.00 (t, *J* = 1.6, 1H, Ar-*H*), 7.96 (dt, *J*_1_ = 7.6, *J*_2_ = 1.6, 1H, Ar-*H*), 7.52 (dt, *J*_1_ = 7.6, *J*_2_ = 1.6, 1H, Ar-*H*), 7.47 (t, *J* = 7.6, 1H, Ar-*H*), 4.71 (s, 2H, N*H*_2_), 4.54 (s, 2H, N*H*_2_), 4.37 (dd, *J*_1_ = 10.8, *J*_2_ = 4.0, 1H, OC*H*_2_), 4.30–4.25 (m, 1H, C*H*), 4.19 (dd, *J*_1_ = 10.8, *J*_2_ = 6.0, 1H, OC*H*_2_), 3.96 (dd, *J*_1_ = 8.4, *J*_2_ = 6.4, 1H, OC*H*_2_), 3.92 (s, 3H, OC*H*_3_), 3.72 (dd, *J*_1_ = 8.4, *J*_2_ = 6.0, 1H, OC*H*_2_), 1.31 (s, 3H, C*H*_3_), 1.25 (s, 3H, C*H*_3_); ES-MS 375.2(M + H)^+^; HRMS Calcd. for C_18_H_23_N_4_O_5_^+^ 375.1668, found 375.1666.

#### General Procedure for the Synthesis of Compounds **10a-q** and **11a-q**

The compounds **8a-q** or **9a-q** (1.42 mmol) was added to 0.25 M H_2_SO_4_ (20 mL) and stirred at room temperature overnight. After the completion of the reaction as indicated by TLC analysis, the reaction solution was adjusted the pH to 9 and extracted with EtOAc (30 mL × 3). The combined organic layers were washed by H_2_O and dried with Na_2_SO_4_, filtered and concentrated. The residue was purified by column chromatography on silica gel using CH_2_Cl_2_/CH_3_OH (80:1, *v*/*v*) as the eluting solvent to the desired compounds.

*(R)-2,4-Diamino-5-(4-chlorophenyl)-6-(1,2-dihydroxypropyl)pyrimidine* (**10a**). White solid 0.37 g, yield 84%; m.p. 164.4–166.3 °C; IR: υ_max_/cm^−1^ 3443 (OH), 3396 (OH), 3335 (NH), 3224 (NH), 1658 (C=N), 1627 (C=N), 1604 (C=C), 1591 (C=C), 1569 (C=C), 1555 (C=C), 1503 (C=C), 1483 (C=C), 796 (C-Cl); ^1^H-NMR (DMSO-*d*_6_) δ 7.38 (d, *J* = 8.4, 2H, Ar-*H*), 7.27 (d, *J* = 8.4, 2H, Ar-*H*), 6.02 (s, 2H, N*H*_2_), 5.63 (s, 2H, N*H*_2_), 4.72 (d, *J* = 4.8, 1H, CHO*H*), 4.50 (t, *J* = 5.8, 1H, CH_2_O*H*), 4.08 (d, *J* = 5.2, 2H, OC*H*_2_), 3.62–3.56 (m, 1H, C*H*), 3.29–3.23 (m, 2H, OC*H*_2_); ES-MS 311.1 (M + H)^+^; HRMS Calcd. for C_13_H_16_ClN_4_O_3_^+^ 311.0911, found 311.0909.

*(R)-2,4-Diamino-5-(4-fluorophenyl)-6-(1,2-dihydroxypropyl)pyrimidine* (**10b**). White solid 0.30 g, yield 68%; m.p. 122.4–122.8 °C; IR: υ_max_/cm^−1^ 3520 (OH), 3472 (OH), 3407 (NH), 3349 (NH), 1651 (C=N), 1617 (C=N), 1592 (C=C), 1570 (C=C), 1484 (C=C), 1397 (C-F); ^1^H-NMR (DMSO-*d*_6_) δ 7.28 (dd, *J*_1_ = 8.8, *J*_2_ = 5.6, 2H, Ar-*H*), 7.15 (t, *J* = 8.8, 2H, Ar-*H*), 5.99 (s, 2H, N*H*_2_), 5.57 (s, 2H, N*H*_2_), 4.72 (d, *J* = 4.8, 1H, CHO*H*), 4.49 (t, *J* = 5.6, 1H, CH_2_O*H*), 4.08 (d, *J* = 5.6, 2H, OC*H*_2_), 3.63–3.55 (m, 1H, C*H*), 3.31–3.24 (m, 2H, OC*H*_2_); ES-MS 295.1 (M + H)^+^; HRMS Calcd. for C_13_H_16_FN_4_O_3_^+^ 295.1206, found 295.1204.

*(R)-2,4-Diamino-5-(3,4-dichlorophenyl)-6-(1,2-dihydroxypropyl)pyrimidine* (**10c**). White solid 0.32 g, yield 71%; m.p. 159.5–160.2 °C; IR: υ_max_/cm^−1^ 3504 (OH), 3476 (OH), 3344 (NH), 3229 (NH), 1650 (C=N), 1619(C=N), 1593 (C=C), 1570 (C=C), 1490 (C=C), 792 (C-Cl); ^1^H-NMR (DMSO-*d*_6_) δ 7.56 (d, *J* = 8.0, 1H, Ar-*H*), 7.47 (d, *J* = 2.0, 1H, Ar-*H*), 7.24 (dd, *J*_1_ =8.0, *J*_2_ = 2.0, 1H, Ar-*H*), 6.07 (s, 2H, N*H*_2_), 5.79 (s, 2H, N*H*_2_), 4.74 (d, 1H, *J* = 4.8, CHO*H*), 4.52 (t, *J* = 5.8, 1H, CH_2_O*H*), 4.14–4.06 (m, 2H, OC*H*_2_), 3.63–3.57 (m, 1H, C*H*), 3.28–3.31 (m, 2H, OC*H*_2_); ES-MS 345.1 (M + H)^+^; HRMS Calcd. for C_13_H_15_Cl_2_N_4_O_3_^+^ 345.0521, found 345.0519.

*(R)-2,4-Diamino-5-(3-chloro-4-fluorophenyl)-6-(1,2-dihydroxypropyl)pyrimidine* (**10d**). White solid 0.39 g, yield 88%; m.p. 158.0–159.9 °C; IR: υ_max_/cm^−1^ 3484 (OH), 3437 (OH), 3318 (NH), 3209 (NH), 1627 (C=N), 1586 (C=C), 1560 (C=C), 1349 (C-F), 801 (C-Cl); ^1^H-NMR (DMSO-*d*_6_) δ 7.40 (dd, *J*_1_ = 7.6, *J*_2_ = 2.4, 1H, Ar-*H*), 7.36 (t, *J* = 9.0, 1H, Ar-*H*), 7.23 (ddd, *J*_1_ = 8.4, *J*_2_ = 4.8, *J*_3_ = 2.0, 1H, Ar-*H*), 6.04 (s, 2H, N*H*_2_), 5.74 (s, 2H, N*H*_2_), 4.74 (d, *J* = 4.8, 1H, CHO*H*), 4.51 (t, *J* = 5.8, 1H, CH_2_O*H*), 4.13–4.05 (m, 2H, OC*H*_2_), 3.63–3.56 (m, 1H, C*H*), 3.30–3.26 (m, 2H, OC*H*_2_); ES-MS 329.1(M + H)^+^; HRMS Calcd. for C_13_H_15_ClFN_4_O_3_^+^ 329.0817, found 329.0820.

*(R)-2,4-Diamino-5-(4-trifluoromethylphenyl)-6-(1,2-dihydroxypropyl)pyrimidine* (**10e**). White solid 0.37 g, yield 83%; m.p. 170.5–171.0 °C; IR: υ_max_/cm^−1^ 3587 (OH), 3506 (OH), 3378 (NH), 3226 (NH), 1631 (C=N), 1591 (C=C), 1559 (C=C), 1520 (C=C), 1322 (C-F); ^1^H-NMR (DMSO-*d*_6_) δ 7.67 (d, *J* = 8.4, 2H, Ar-*H*), 7.50 (d, *J* = 8.0, 2H, Ar-*H*), 6.09 (s, 2H, N*H*_2_), 5.75 (s, 2H, N*H*_2_), 4.74 (d, *J* = 5.2, 1H, CHO*H*), 4.52 (t, *J* = 5.8, 1H, CH_2_O*H*), 4.15–4.07 (m, 2H, OC*H*_2_), 3.64–3.57 (m, 1H, C*H*), 3.24–3.30 (m, 2H, OC*H*_2_); ES-MS 345.1 (M + H)^+^; HRMS Calcd. for C_14_H_16_F_3_N_4_O_3_^+^ 345.1175, found 345.1173.

*(R)-2,4-Diamino-5-(3-trifluoromethylphenyl)-6-(1,2-dihydroxypropyl)pyrimidine* (**10f**). White solid 0.41 g, yield 92%; m.p. 137.8–138.9 °C; IR: υ_max_/cm^−1^ 3521 (OH), 3474 (OH), 3349 (NH), 3235 (NH), 1650 (C=N), 1621 (C=N), 1594 (C=C), 1569 (C=C), 1495 (C=C), 1341 (C-F); ^1^H-NMR (DMSO-*d*_6_) δ 7.58–7.55 (m, 4H, Ar-*H*), 6.08 (s, 2H, N*H*_2_), 5.74 (s, 2H, N*H*_2_), 4.72 (d, 1H, *J* = 5.2, CHO*H*), 4.50 (t, *J* = 5.8, 1H, CH_2_O*H*), 4.11 (d, *J* = 4.8, 2H, OC*H*_2_), 3.62–3.56 (m, 1H, C*H*), 3.31–3.26 (m, 2H, OC*H*_2_); ES-MS 345.1 (M + H)^+^; HRMS Calcd. for C_14_H_16_F_3_N_4_O_3_^+^ 345.1175, found 345.1172.

*(R)-2,4-Diamino-5-(4-trifluoromethoxyphenyl)-6-(1,2-dihydroxypropyl)pyrimidine* (**10g**). White solid 0.42 g, yield 85%; m.p. 181.4–182.1 °C; IR: υ_max_/cm^−1^ 3579 (OH), 3457 (OH), 3341(NH), 3231 (NH), 1636 (C=N), 1590 (C=C), 1557 (C=C), 1549 (C=C), 1501 (C=C), 1316 (C-F); ^1^H-NMR (DMSO-*d*_6_) δ 7.38 (d, *J* = 8.4, 2H, Ar-*H*), 7.31 (d, *J* = 8.4, 2H, Ar-*H*), 6.06 (s, 2H, N*H*_2_), 5.69 (s, 2H, N*H*_2_), 4.77 (d, *J* = 5.2, 1H, CHO*H*), 4.53 (t, *J* = 5.8, 1H, CH_2_O*H*), 4.10 (d, *J* = 5.2, 2H, OC*H*_2_), 3.63–3.57 (m, 1H, C*H*), 3.32–3.23 (m, 2H, OC*H*_2_); ES-MS 361.1 (M + H)^+^; HRMS Calcd. for C_14_H_16_F_3_N_4_O_4_^+^ 361.1124, found 361.1120.

*(R)-2,4-Diamino-5-(3-trifluoromethoxyphenyl)-6-(1,2-dihydroxypropyl)pyrimidine* (**10h**). White solid 0.44 g, yield 89%; m.p. 58.5–59.6 °C; IR: υ_max_/cm^−1^ 3467 (OH), 3379 (OH), 3350 (NH), 1612 (C=N), 1562 (C=C), 1495 (C=C), 1348 (C-F); ^1^H-NMR (DMSO-*d*_6_) δ 7.46 (t, *J* = 8.0, 2H, Ar-*H*), 7.31 (d, *J* = 8.0, 2H, Ar-*H*), 7.23 (s, 1H, Ar-*H*), 7.20 (d, *J* = 8.0, 1H, Ar-H), 6.09 (s, 2H, N*H*_2_), 5.73 (s, 2H, N*H*_2_), 4.75 (d, *J* = 4.8, 1H, CHO*H*), 4.52 (t, *J* = 5.6, 1H, CH_2_O*H*), 4.10 (d, *J* = 5.2, 2H, OC*H*_2_), 3.63–3.56 (m, 1H, C*H*), 3.31–3.24 (m, 2H, OC*H*_2_); ES-MS 361.1 (M + H)^+^; HRMS Calcd. for C_14_H_16_F_3_N_4_O_4_^+^ 361.1124, found 361.1128.

*(R)-2,4-Diamino-5-(4-cyanophenyl)-6-(1,2-dihydroxypropyl)pyrimidine* (**10i**). White solid 0.39 g, yield 88%; m.p. 199.7–200.8 °C; IR: υ_max_/cm^−1^ 3459 (OH), 3382 (NH), 2228(CN), 1658 (C=N), 1624(C=N), 1592 (C=C), 1563 (C=C), 1549 (C=C), 1510 (C=C); ^1^H-NMR (DMSO-*d*_6_) δ 7.77 (d, *J* = 8.4, 2H, Ar-*H*), 7.48 (d, *J* = 8.0, 2H, Ar-*H*), 6.15 (s, 2H, N*H*_2_), 5.82 (s, 2H, N*H*_2_), 4.77 (d, *J* = 4.8, 1H, CHO*H*), 4.55 (t, *J* = 5.6, 1H, CH_2_O*H*), 4.14–4.06 (m, 2H, OC*H*_2_), 3.63–3.57 (m, 1H, C*H*), 3.31–3.25 (m, 2H, OC*H*_2_); ES-MS 302.1 (M + H)^+^; HRMS Calcd. for C_14_H_16_N_5_O_3_^+^ 302.1253, found 302.1250.

*(R)-2,4-Diamino-5-(4-methoxyphenyl)-6-(1,2-dihydroxypropyl)pyrimidine* (**10j**). White solid 0.37 g, yield 84%; m.p. 188.4–188.9 °C; IR: υ_max_/cm^−1^ 3544 (OH), 3497 (OH), 3466 (NH), 3339 (NH), 1639 (C=N), 1621 (C=N), 1591 (C=C), 1557 (C=C); ^1^H-NMR (DMSO-*d*_6_) δ 7.16 (d, *J* = 8.8, 2H, Ar-*H*), 6.92 (d, *J* = 8.8, 2H, Ar-*H*), 5.94 (s, 2H, N*H*_2_), 5.46 (s, 2H, N*H*_2_), 4.73 (d, 1H, *J* = 4.8, CHO*H*), 4.49 (t, *J* = 5.8, 1H, CH_2_O*H*), 4.11–4.04 (m, 2H, OC*H*_2_), 3.76 (s, 3H, OC*H*_3_), 3.62–3.55 (m, 1H, C*H*), 3.33–3.24 (m, 2H, OC*H*_2_); ES-MS 307.1 (M + H)^+^; HRMS Calcd. for C_14_H_19_N_4_O_4_^+^ 307.1406, found 307.1404.

*(R)-2,4-Diamino-5-(3,4,5-trimethoxyphenyl)-6-(1,2-dihydroxypropyl)pyrimidine* (**10k**). White solid 0.34 g, yield 75%; m.p. 198.6–199.7 °C; IR: υ_max_/cm^−1^ 3489 (OH), 3439 (OH), 3356 (NH), 3208 (NH), 1626 (C=N), 1589 (C=C), 1569 (C=C), 1489 (C=C); ^1^H-NMR (DMSO-*d*_6_) δ 6.55 (s, 2H, Ar-*H*), 5.96 (s, 2H, N*H*_2_), 5.69 (s, 2H, N*H*_2_), 4.76 (d, *J* = 5.2, 1H, CHO*H*), 4.53 (t, *J* = 5.6, 1H, CH_2_O*H*), 4.16–4.05 (m, 2H, OC*H*_2_), 3.75 (s, 6H, OC*H*_3_ × 2), 3.68 (s, 3H, OC*H*_3_), 3.67–3.60 (m, 1H, C*H*), 3.38–3.28 (m, 2H, OC*H*_2_); ES-MS 367.2 (M + H)^+^; HRMS Calcd. for C_16_H_23_N_4_O_6_^+^ 367.1618, found 367.1616.

*(R)-2,4-Diamino-5-[3-(2,2,2-trifluoroethoxymethyl)phenyl]-6-(1,2-dihydroxypropyl)pyrimidine* (**10l**). White solid 0.41 g, yield 82%; m.p. 48.2–49.3 °C; IR: υ_max_/cm^−1^ 3478 (OH), 3347 (NH), 3215 (NH), 1612 (C=N), 1562 (C=C), 1492 (C=C), 1348(C-F); ^1^H-NMR (DMSO-*d*_6_) δ 7.36 (t, *J* = 7.8, 1H, Ar-*H*), 7.20–7.23 (m,3H, Ar-*H*), 6.02 (s, 2H, N*H*_2_), 5.59 (s, 2H, N*H*_2_), 4.74 (d, *J* = 5.2, 1H, CHO*H*), 4.65 (s, 2H, C*H*_2_), 4.51 (t, *J* = 5.6, 1H, CH_2_O*H*), 4.09 (dd, *J*_1_ = 18.8 , *J*_2_ = 9.6 , 2H, CF_3_C*H*_2_), 4.08 (d, *J* = 5.2, 2H, OC*H*_2_), 3.62–3.55 (m, 1H, C*H*), 3.34–3.23 (m, 2H, OC*H*_2_); ES-MS 389.1(M + H)^+^; HRMS Calcd. for C_16_H_20_F_3_N_4_O_4_^+^ 389.1437, found 389.1435.

*(R)-2,4-Diamino-5-[3,5-dimethyl-4-(N-methoxyaminosulfonyl)phenyl]-6-(1,2-dihydroxypropyl)pyrimidine* (**10m**). White solid 0.40 g, yield 88%; m.p. 98.3–99.3 °C; IR: υ_max_/cm^−1^ 3475 (OH), 3378 (NH), 3216 (NH), 1610 (C=N), 1594 (C=C), 1562 (C=C), 1482(C=C); ^1^H-NMR (DMSO-*d*_6_) δ 10.29 (s, 1H, N*H*), 7.18 (s, 2H, Ar-*H*), 6.12 (s, 2H, N*H*_2_), 5.80 (s, 2H, N*H*_2_), 4.79 (d, *J* = 4.8, 1H, CHO*H*), 4.56 (t, *J* = 5.8, 1H, CH_2_O*H*), 4.15–4.07 (m, 2H, OC*H*_2_), 3.64–3.58 (m, 1H, C*H* and OC*H*_3_), 3.33–3.27 (m, 2H, OC*H*_2_), 2.59 (s, 6H, C*H*_3_); ES-MS 414.1(M + H)^+^; HRMS Calcd. for C_16_H_24_N_5_O_6_S^+^ 414.1447, found 414.1450.

*(R)-2,4-Diamino-5-[4-(morpholine-4-carbonyl)phenyl]-6-(1,2-dihydroxypropyl)pyrimidine* (**10n**). White solid 0.53 g, yield 90%; m.p. 210.1–211.3 °C; IR (KBr): υ_max_/cm^−1^ 3446 (OH), 3343 (NH), 3220 (NH), 1630 (C=N), 1606 (C=C), 1591 (C=C), 1569 (C=C), 1511 (C=C); ^1^H-NMR (DMSO-*d*_6_) δ 7.36 (d, *J* = 8.4, 2H, Ar-*H*), 7.32 (d, *J* = 8.4, 2H, Ar-*H*), 6.04 (s, 2H, N*H*_2_), 5.69 (s, 2H, N*H*_2_), 4.77 (d, *J* = 5.2, 1H, CHO*H*), 4.53 (t, *J* = 5.6, 1H, CH_2_O*H*), 4.10 (d, *J* = 5.6, 2H, OC*H*_2_), 3.63–3.59 (m, 1H, C*H*), 3.76–3.42 (m, 8H, C*H*_2_ × 4), 3.30–3.27 (m, 2H, OC*H*_2_); ES-MS 390.2 (M + H)^+^; HRMS Calcd. for C_18_H_24_N_5_O_5_^+^ 390.1777, found 390.1774.

*(R)-2,4-Diamino-5-[4-(3-trifluoromethoxyanilinocarbonyl)phenyl]-6-(1,2-dihydroxypropyl)pyrimidine* (**10o**). White solid 0.46 g, yield 91%; m.p. 142.0–143.6 °C; IR: υ_max_/cm^−1^ 3346 (OH), 3228 (NH), 1656 (C=N), 1632 (C=N), 1607 (C=C), 1549 (C=C), 1490 (C=C), 1349(C-F); ^1^H-NMR (DMSO-*d*_6_) δ 10.49 (s, 1H, N*H*), 7.98–7.96 (m, 3H, Ar-*H*), 7.80 (dd, *J*_1_ = 8.0, *J*_2_ = 1.2, 1H, Ar-*H*), 7.51–7.45 (m, 3H, Ar-*H*), 7.09 (dt, *J*_1_ = 8.4, *J*_2_ = 1.2, 1H, Ar-*H*), 6.10 (s, 2H, N*H*_2_), 5.71 (s, 2H, N*H*_2_), 4.77 (d, *J* = 5.2, 1H, CHO*H*), 4.55 (t, *J* = 5.6, 1H, CH_2_O*H*), 4.12 (d, *J* = 5.2, 2H, OC*H*_2_), 3.65–3.59 (m, 1H, C*H*), 3.33–3.26 (m, 2H, OC*H*_2_); ES-MS 480.1 (M + H)^+^; HRMS Calcd. for C_21_H_21_F_3_N_5_O_5_^+^ 480.1495, found 480.1492.

*(R)-2,4-Diamino-5-(4-methoxycarbonylphenyl)-6-(1,2-dihydroxypropyl)pyrimidine* (**10p**). White solid 0.40 g, yield 90%; m.p. 186.7–188.2 °C; IR: υ_max_/cm^−1^ 3449 (OH), 3330 (NH), 3219 (NH), 1705 (C=O), 1631 (C=N), 1592 (C=C), 1514 (C=C); ^1^H-NMR (DMSO-*d*_6_) δ 7.91 (d, *J* = 8.4, 2H, Ar-*H*), 7.44 (d, *J* = 8.4, 2H, Ar-*H*), 6.10 (s, 2H, N*H*_2_), 5.73 (s, 2H, N*H*_2_), 4.75 (d, *J* = 4.8, 1H, CHO*H*), 4.53 (t, *J* = 5.8, 1H, CH_2_O*H*), 4.09 (d, *J* = 5.2, 2H, OC*H*_2_), 3.86 (s, 3H, OC*H*_3_), 3.63–3.55 (m, 1H, C*H*), 3.30–3.25 (m, 2H, OC*H*_2_); ES-MS 335.1 (M + H)^+^; HRMS Calcd. for C_15_H_19_N_4_O_5_^+^ 335.1355, found 335.1350.

*(R)-2,4-Diamino-5-(3-methoxycarbonylphenyl)-6-(1,2-dihydroxypropyl)pyrimidine* (**10q**). White solid 0.40 g, yield 90%; m.p. 140.6–141.5 °C; IR: υ_max_/cm^−1^ 3457 (OH), 3336 (NH), 3224 (NH), 1705 (C=O), 1658 (C=N), 1600 (C=C), 1560 (C=C), 1490 (C=C); ^1^H-NMR (DMSO-*d*_6_) δ 7.84 (t, *J* = 1.2, 1H, Ar-*H*), 7.81 (dt, *J*_1_ = 7.6, *J*_2_= 1.6, 1H, Ar-*H*), 7.54 (dt, *J*_1_ = 8.0, *J*_2_ = 1.6, 1H, Ar-*H*), 7.49 (t, *J* = 7.6, 1H, Ar-*H*), 6.06 (s, 2H, N*H*_2_), 5.68 (s, 2H, N*H*_2_), 4.72 (d, *J* = 5.2, 1H, CHO*H*), 4.50 (t, *J* = 5.6, 1H, CH_2_O*H*), 4.09 (d, *J* = 5.6, 2H, OC*H*_2_), 3.85 (s, 3H, OC*H*_3_), 3.61–3.54 (m, 1H, C*H*), 3.30–3.24 (m, 2H, OC*H*_2_); ES-MS 335.1 (M + H)^+^; HRMS Calcd. for C_15_H_19_N_4_O_5_^+^ 335.1355, found 335.1358.

*(S)-2,4-Diamino-5-(4-chlorophenyl)-6-(1,2-dihydroxypropyl)pyrimidine* (**11a**). White solid 0.36 g, yield 85%; m.p. 169.8–169.9 °C; IR: υ_max_/cm^−1^ 3446 (OH), 3396 (OH), 3336 (NH), 3214 (NH), 1606 (C=N), 1589 (C=C), 1569 (C=C), 1556 (C=C), 1503(C=C), 797 (C-Cl); ^1^H-NMR (DMSO-*d*_6_) δ 7.38 (d, *J* = 8.4, 2H, Ar-*H*), 7.27 (d, *J* = 8.4, 2H, Ar-*H*), 6.03 (s, 2H, N*H*_2_), 5.65 (s, 2H, N*H*_2_), 4.74 (d, *J* = 5.2, 1H, CHO*H*), 4.51 (t, *J* = 5.6, 1H, CH_2_O*H*), 4.08 (d, *J* = 5.6, 2H, OC*H*_2_), 3.63–3.56 (m, 1H, C*H*), 3.31–3.23 (m, 2H, OC*H*_2_); ES-MS 311.1 (M + H)^+^; HRMS Calcd. for C_13_H_16_ClN_4_O_3_^+^ 311.0911, found 311.0908.

*(S)-2,4-Diamino-5-(4-fluorophenyl)-6-(1,2-dihydroxypropyl)pyrimidine* (**11b**). White solid 0.42 g, yield 95%; m.p. 111.7–113.7 °C; IR: υ_max_/cm^−1^ 3482 (OH), 3343 (NH), 3212 (NH), 1614 (C=N), 1593 (C=C), 1562 (C=C), 1509 (C=C), 1398 (C-F); ^1^H-NMR (DMSO-*d*_6_) δ 7.27 (dd, *J*_1_ = 8.8, *J*_2_ = 6.0, 2H, Ar-*H*), 7.16 (t, *J* = 8.8, 2H, Ar-*H*), 6.00 (s, 2H, N*H*_2_), 5.57 (s, 2H, N*H*_2_), 4.73 (d, *J* = 5.2, 1H, CHO*H*), 4.50 (t, *J* = 5.8, 1H, CH_2_O*H*), 4.08 (d, *J* = 5.2, 2H, OC*H*_2_), 3.62–3.55 (m, 1H, C*H*), 3.33–3.23 (m, 2H, OC*H*_2_); ES-MS 295.1 (M + H)^+^; HRMS Calcd. for C_13_H_16_FN_4_O_3_^+^ 295.1206, found 295.1208.

*(S)-2,4-Diamino-5-(3,4-dichlorophenyl)-6-(1,2-dihydroxypropyl)pyrimidine* (**11c**). White solid 0.35 g, yield 87%; m.p. 160.6–160.8 °C; IR: υ_max_/cm^−1^ 3504 (OH), 3476 (OH), 3383 (NH), 3346 (NH), 1649 (C=N), 1617 (C=N), 1592 (C=C), 1569 (C=C), 1491 (C=C), 793 (C-Cl); ^1^H-NMR (DMSO-*d*_6_) δ 7.56 (d, *J* = 8.0, 2H, Ar-*H*), 7.47 (d, *J* = 2.0, 1H, Ar-*H*), 7.24 (dd, *J*_1_ = 8.0, *J*_2_ = 2.0, 1H, Ar-*H*), 6.08 (s, 2H, N*H*_2_), 5.81 (s, 2H, N*H*_2_), 4.75 (d, 1H, *J* = 5.2, CHO*H*), 4.53 (t, *J* = 5.6, 1H, CH_2_O*H*), 4.14–4.06 (m, 2H, OC*H*_2_), 3.63–3.57 (m, 1H, C*H*), 3.31–3.25 (m, 2H, OC*H*_2_); ES-MS 345.1 (M + H)^+^; HRMS Calcd. for C_13_H_15_Cl_2_N_4_O_3_^+^ 345.0521, found 345.0523.

*(S)-2,4-Diamino-5-(3-chloro-4-fluorophenyl)-6-(1,2-dihydroxypropyl)pyrimidine* (**11d**). White solid 0.37 g, yield 90%; m.p. 157.7–158.1 °C; IR: υ_max_/cm^−1^ 3484 (OH), 3329 (NH), 3210 (NH), 1622 (C=N), 1605 (C=C), 1590 (C=C), 1561 (C=C), 1347 (C-F), 799 (C-Cl); ^1^H-NMR (DMSO-*d*_6_) δ 7.40 (dd, *J*_1_ = 7.2, *J*_2_ = 2.0, 1H, Ar-*H*), 7.36 (t, *J* = 8.8, 1H, Ar-*H*), 7.25–7.21 (ddd, *J*_1_ = 8.8, *J*_2_ = 4.8, *J*_3_ = 2.0, 1H, Ar-*H*), 6.05 (s, 2H, N*H*_2_), 5.74 (s, 2H, N*H*_2_), 4.74 (d, *J* = 5.2, 1H, CHO*H*), 4.52 (t, *J* = 5.6, 1H, CH_2_O*H*), 4.13–4.06 (m, 2H, OC*H*_2_), 3.63–3.56 (m, 1H, C*H*), 3.30–3.26 (m, 2H, OC*H*_2_); ES-MS 329.1(M + H)^+^; HRMS Calcd. for C_13_H_15_ClFN_4_O_3_^+^ 329.0817, found 329.0821.

*(S)-2,4-Diamino-5-(4-trifluoromethylphenyl)-6-(1,2-dihydroxypropyl)pyrimidine* (**11e**). White solid 0.37 g, yield 83%; m.p. 170.8–171.5 °C; IR: υ_max_/cm^−1^ 3620 (OH), 3512 (OH), 3334 (NH), 3225 (NH), 1661 (C=N), 1632 (C=N), 1598 (C=C), 1554 (C=C), 1493 (C=C), 1339 (C-F); ^1^H-NMR (DMSO-*d*_6_) δ 7.67 (d, *J* = 8.0, 2H, Ar-*H*), 7.50 (d, *J* = 8.0, 2H, Ar-*H*), 6.10 (s, 2H, N*H*_2_), 5.77 (s, 2H, N*H*_2_), 4.75 (d, *J* = 5.2, 1H, CHO*H*), 4.53 (t, *J* = 5.6, 1H, CH_2_O*H*), 4.15–4.07 (m, 2H, OC*H*_2_), 3.64–3.57 (m, 1H, C*H*), 3.31–3.26 (m, 2H, OC*H*_2_); ES-MS 345.1 (M + H)^+^; HRMS Calcd. for C_14_H_16_F_3_N_4_O_3_^+^ 345.1175, found 345.1180.

*(S)-2,4-Diamino-5-(3-trifluoromethylphenyl)-6-(1,2-dihydroxypropyl)pyrimidine* (**11f**). White solid 0.37 g, yield 83%; m.p. 144.2–144.3 °C; IR: υ_max_/cm^−1^ 3474 (OH), 3389 (OH), 3348 (NH), 3224 (NH), 1617 (C=N), 1594 (C=C), 1564 (C=C), 1496 (C=C), 1336 (C-F); ^1^H-NMR (DMSO-*d*_6_) δ 7.59–7.55 (m, 4H, Ar-*H*), 6.09 (s, 2H, N*H*_2_), 5.74 (s, 2H, N*H*_2_), 4.72 (d, *J* = 4.8, 1H, CHO*H*), 4.50 (t, *J* = 5.8, 1H, CH_2_O*H*), 4.12 (d, *J* = 5.2, 2H, OC*H*_2_), 3.63–3.54 (m, 1H, C*H*), 3.31–3.25 (m, 2H, OC*H*_2_); ES-MS 345.1 (M + H)^+^; HRMS Calcd. for C_14_H_16_F_3_N_4_O_3_^+^ 345.1175, found 345.1177.

*(S)-2,4-Diamino-5-(4-trifluoromethoxyphenyl)-6-(1,2-dihydroxypropyl)pyrimidine* (**11g**). White solid 0.44 g, yield 90%; m.p. 141.7–143.2 °C; IR: υ_max_/cm^−1^ 3345 (OH), 3216 (NH), 1614 (C=N), 1595 (C=C), 1561 (C=C), 1349 (C-F); ^1^H-NMR (DMSO-d_6_) δ 7.39 (d, *J* = 8.4, 2H, Ar-*H*), 7.31 (d, *J* = 8.4, 2H, Ar-*H*), 6.05 (s, 2H, N*H*_2_), 5.68 (s, 2H, N*H*_2_), 4.76 (d, *J* = 4.8, 1H, CHO*H*), 4.52 (t, *J* = 5.6, 1H, CH_2_O*H*), 4.10 (d, *J* = 5.2, 2H, OC*H*_2_), 3.64–3.57 (m, 1H, C*H*), 3.31–3.24 (m, 2H, OC*H*_2_); ES-MS 361.1 (M + H)^+^; HRMS Calcd. for C_14_H_16_F_3_N_4_O_4_^+^ 361.1124, found 361.1127.

*(S)-2,4-Diamino-5-(3-trifluoromethoxyphenyl)-6-(1,2-dihydroxypropyl)pyrimidine* (**11h**). White solid 0.44 g, yield 90%; m.p. 120.5–122.0 °C; IR: υ_max_/cm^−1^ 3621 (OH), 3340 (OH), 3224 (NH), 1656 (C=N), 1629 (C=N), 1609 (C=C), 1563 (C=C), 1499 (C=C), 1354 (C-F); ^1^H-NMR (DMSO-*d*_6_) δ 7.46 (t, *J* = 7.8, 2H, Ar-*H*), 7.31 (d, *J* = 7.8, 2H, Ar-*H*), 7.23 (s, 1H, Ar-*H*), 7.19 (d, *J* = 7.8, 1H, Ar-*H*), 6.09 (s, 2H, N*H*_2_), 5.73 (s, 2H, N*H*_2_), 4.74 (d, *J* = 4.8, 1H, CHO*H*), 4.51 (t, *J* = 5.6, 1H, CH_2_O*H*), 4.10 (d, *J* = 5.2, 2H, OC*H*_2_), 3.63–3.56 (m, 1H, C*H*), 3.31–3.25 (m, 2H, OC*H*_2_); ES-MS 361.1 (M + H)^+^; HRMS Calcd. for C_14_H_16_F_3_N_4_O_4_^+^ 361.1124, found 361.1122.

*(S)-2,4-Diamino-5-(4-cyanophenyl)-6-(1,2-dihydroxypropyl)pyrimidine* (**11i**). White solid 0.38 g, yield 86%; m.p. 201.6–203.0 °C; IR: υ_max_/cm^−1^ 3458 (OH), 3359 (NH), 2230 (CN), 1631 (C=N), 1604 (C=C) 1586 (C=C), 1563 (C=C), 1550 (C=C); ^1^H-NMR (DMSO-*d*_6_) δ 7.77 (d, *J* = 8.8, 2H, Ar-*H*), 7.48 (d, *J* = 8.8, 2H, Ar-*H*), 6.15 (s, 2H, N*H*_2_), 5.83 (s, 2H, N*H*_2_), 4.76 (d, *J* = 5.2, 1H, CHO*H*), 4.54 (t, *J* = 5.6, 1H, CH_2_O*H*), 4.14–4.07 (m, 2H, OC*H*_2_), 3.62–3.58 (m, 1H, C*H*), 3.31–3.3124 (m, 2H, OC*H*_2_); ES-MS 302.1 (M + H)^+^; HRMS Calcd. for C_14_H_16_N_5_O_3_^+^ 302.1253, found 302.1251.

*(S)-2,4-Diamino-5-(4-methoxyphenyl)-6-(1,2-dihydroxypropyl)pyrimidine* (**11j**). White solid 0.36 g, yield 91%; m.p. 187.4–188.7 °C; IR: υ_max_/cm^−1^ 3542 (OH), 3496 (OH), 3467 (NH), 3339 (NH), 1639 (C=N), 1621 (C=N), 1608 (C=C), 1591 (C=C), 1558 (C=C); ^1^H-NMR (DMSO-*d*_6_) δ 7.15 (d, *J* = 8.8, 2H, Ar-*H*), 6.92 (d, *J* = 8.8, 2H, Ar-*H*), 5.94 (s, 2H, N*H*_2_), 5.46 (s, 2H, N*H*_2_), 4.73 (d, *J* = 4.8, 1H, CHO*H*), 4.50 (t, *J* = 5.6, 1H, CH_2_O*H*), 4.11–4.04 (m, 2H, OC*H*_2_), 3.76 (s, 3H, OC*H*_3_), 3.62–3.56 (m, 1H, CH), 3.32–3.25 (m, 2H, OCH_2_); ES-MS 307.1 (M + H)^+^; HRMS Calcd. for C_14_H_19_N_4_O_4_^+^ 307.1406, found 307.1408.

*(S)-2,4-Diamino-5-(3,4,5-trimethoxyphenyl)-6-(1,2-dihydroxypropyl)pyrimidine* (**11k**). White solid 0.40 g, yield 89%; m.p. 199.1–200.3 °C; IR: υ_max_/cm^−1^ 3489 (OH), 3439 (OH), 3356 (NH), 3208 (NH), 1626 (C=N), 1589 (C=C), 1569 (C=C), 1489 (C=C); ^1^H-NMR (DMSO-*d*_6_) δ 6.55 (s, 2H, Ar-*H*), 5.96 (s, 2H, N*H*_2_), 5.64 (s, 2H, N*H*_2_), 4.76 (d, *J* = 4.8, 1H, CHO*H*), 4.53 (t, *J* = 5.8, 1H, CH_2_O*H*), 4.14–4.07 (m, 2H, OC*H*_2_), 3.75 (s, 6H, OC*H*_3_), 3.68 (s, 3H, OC*H*_3_), 3.65–3.60 (m, 1H, C*H*), 3.38–3.28 (m, 2H, OC*H*_2_); ES-MS 367.2 (M + H)^+^; HRMS Calcd. for C_16_H_23_N_4_O_6_^+^ 367.1618, found 367.1620.

*(S)-2,4-Diamino-5-[3-(2,2,2-trifluoroethoxymethyl)phenyl]-6-(1,2-dihydroxypropyl)pyrimidine* (**11l**). White solid 0.38 g, yield 84%; m.p. 42.7–43.5 °C; IR: υ_max_/cm^−1^ 3477 (OH), 3346 (NH), 3215 (NH), 1613 (C=N), 1561 (C=C), 1495 (C=C), 1346 (C-F); ^1^H-NMR (DMSO-*d*_6_) δ 7.36 (t, *J* = 7.8, 1H, Ar-*H*), 7.23–7.20 (m,3H, Ar-*H*), 6.01 (s, 2H, N*H*_2_), 5.57 (s, 2H, N*H*_2_), 4.73 (d, *J* = 5.2, 1H, CHO*H*), 4.66 (s, 2H, OC*H*_2_), 4.50 (t, *J* = 5.6, 1H, CH_2_O*H*), 4.13–4.06 (m, 4H, OC*H*_2_and CF_3_C*H*_2_), 3.62–3.55 (m, 1H, C*H*), 3.31–3.23 (m, 2H, OC*H*_2_); ES-MS 389.1(M + H)^+^; HRMS Calcd. for C_16_H_20_F_3_N_4_O_4_^+^ 389.1437, found 389.1440.

*(S)-2,4-Diamino-5-[3,5-dimethyl-4-(N-methoxyaminosulfonyl)phenyl]-6-(1,2-dihydroxypropyl)pyrimidine* (**11m**). White solid 0.31 g, yield 85%; m.p. 93.0–93.8 °C; IR: υ_max_/cm^−1^ 3463 (OH), 3379 (NH), 3215 (NH), 1612 (C=N), 1594 (C=C), 1563 (C=C); ^1^H-NMR (DMSO-*d*_6_) δ 10.29 (s, 1H, N*H*), 7.18 (s, 2H, Ar-*H*), 6.11 (s, 2H, N*H*_2_), 5.78 (s, 2H, N*H*_2_), 4.77 (d, *J* = 5.2, 1H, CHO*H*), 4.55 (t, *J* = 5.6, 1H, CH_2_O*H*), 4.15–4.07 (m, 2H, OC*H*_2_), 3.62–3.58 (m, 4H, OC*H*_3_ and C*H*), 3.32–3.28 (m, 2H, OC*H*_2_), 2.59 (s, 6H, C*H*_3_ × 2); ES-MS 414.1(M + H)^+^; HRMS Calcd. for C_16_H_24_N_5_O_6_S^+^ 414.1447, found 414.1449.

*(S)-2,4-Diamino-5-[4-(morpholino-4-carbonyl)phenyl]-6-(1,2-dihydroxypropyl)pyrimidine* (**11n**). White solid 0.35 g, yield 86%; m.p. 209.6–210.2 °C; IR: υ_max_/cm^−1^ 3443 (OH), 3342 (NH), 3220 (NH), 1629 (C=N), 1609 (C=C), 1590 (C=C), 1568 (C=C), 1511 (C=C); ^1^H-NMR (DMSO-*d*_6_) δ 7.36 (d, *J* = 8.4, 2H, Ar-*H*), 7.34 (d, *J* = 8.4, 2H, Ar-*H*), 6.04 (s, 2H, N*H*_2_), 5.69 (s, 2H, N*H*_2_), 4.77 (d, *J* = 5.2, 1H, CHO*H*), 4.53 (t, *J* = 5.6, 1H, CH_2_O*H*), 4.10 (d, *J* = 5.2, 2H, OC*H*_2_), 3.63–3.59 (m, 1H, C*H*), 3.74–3.38 (m, 8H, C*H*_2_ × 4), 3.32–3.25 (m, 2H, OC*H*_2_); ES-MS 390.2 (M + H)^+^**;** HRMS Calcd. for C_18_H_24_N_5_O_5_^+^ 390.1777, found 390.1779.

*(S)-2,4-Diamino-5-[4-(3-trifluoromethoxyanilinocarbonyl)phenyl]-6-(1,2-dihydroxypropyl)pyrimidine* (**11o**). White soilid 0.44 g, 87%; m.p. 143.2–145.0 °C; IR: υ_max_/cm^−1^ 3336 (OH), 3225 (NH), 1658 (C=N), 1606 (C=C), 1549 (C=C), 1491 (C=C), 1350 (C-F); ^1^H-NMR (DMSO-*d*_6_) δ 10.49 (s, 1H, N*H*), 7.98–7.96 ( m, 3H, Ar-*H*), 7.80 (dd, *J*_1_ = 8.2, *J*_2_ = 1.0, 1H, Ar-*H*), 7.51–7.45 (m, 3H, Ar-*H*), 7.09 (dt, *J*_1_ = 8.4, *J*_2_= 1.2, 1H, Ar-*H*), 6.10 (s, 2H, N*H*_2_), 5.70 (s, 2H, N*H*_2_), 4.77 (d, *J* = 5.2, 1H, CHO*H*), 4.55 (t, *J* = 5.8, 1H, CH_2_O*H*), 4.12 (d, *J* = 5.2, 2H, C*H*_2_), 3.58–3.65 (m, 1H, C*H*), 3.28–3.33 (m, 2H, O*H*); ES-MS 480.1 (M + H)^+^; HRMS Calcd. for C_21_H_21_F_3_N_5_O_5_^+^ 480.1495, found 480.1497.

*(S)-2,4-Diamino-5-(4-methoxycarbonylphenyl)-6-(1,2-dihydroxypropyl)pyrimidine* (**11p**). White solid 0.32 g, yield 90%; m.p. 211.3–212.4 °C; IR: υ_max_/cm^−1^ 3562 (OH), 3450 (NH), 3227 (NH), 3217 (NH), 1712 (C=O), 1629 (C=N), 1592 (C=C), 1573 (C=C), 1555 (C=C), 1515 (C=C); ^1^H-NMR (DMSO-*d*_6_) δ 7.92 (d, *J* = 8.4, 2H, Ar-*H*), 7.44 (d, *J* = 8.4, 2H, Ar-*H*), 6.10 (s, 2H, N*H*_2_), 5.74 (s, 2H, N*H*_2_), 4.74 (d, *J* = 5.2, 1H, CHO*H*), 4.52 (t, *J* = 5.6, 1H, CH_2_O*H*), 4.13–4.06 (m, 2H, OC*H*_2_), 3.86 (s, 3H, OC*H*_3_) 3.6–3.57 (m, 1H, C*H*), 3.3–3.24 (m, 2H, OC*H*_2_); ES-MS 335.1 (M + H)^+^; HRMS Calcd. for C_15_H_19_N_4_O_5_^+^ 335.1355, found 335.1357.

*(S)-2,4-Diamino-5-(3-methoxycarbonylphenyl)-6-(1,2-dihydroxypropyl)pyrimidine* (**11q**). White solid 0.38 g, yield 85%; m.p. 181.4–182.3 °C; IR: υ_max_/cm^−1^ 3577 (OH), 3525 (OH), 3453 (NH), 3338 (NH), 1705 (C=O), 1637 (C=N), 1590 (C=C), 1558 (C=C), 1501 (C=C); ^1^H-NMR (DMSO-*d*_6_) δ 7.84 (s, 1H, Ar-*H*), 7.81 (d, *J* = 7.2, 1H, Ar-*H*) 7.54 (d, *J* = 7.6, 1H, Ar-*H*), 7.49 (t, *J* = 7.6, 1H, Ar-*H*), 6.06 (s, 2H, N*H*_2_), 5.69 (s, 2H, N*H*_2_), 4.72 (d, *J* = 4.8, 1H, CHO*H*), 4.50 (t, *J* = 5.8, 1H, CH_2_O*H*), 4.09 (d, *J* = 5.2, 2H, OC*H*_2_), 3.85 (s, 3H, OC*H*_3_) 3.61–3.54 (m, 1H, C*H*), 3.30–3.22 (m, 2H, OC*H*_2_); ES-MS 335.1 (M + H)^+^; HRMS Calcd. for C_15_H_19_N_4_O_5_^+^ 335.1355, found 335.1353.

#### General Procedure for the Synthesis of 2,4-Diamino-6-substituted Pyrimidine Derivatives **13a-d**

Under argon, to a solution of substituted methanol **12a-d** (17.45 mmol) in dry DMSO or THF (30 mL) was added NaH (60%, 21.82 mmol) and stirred at room temperature for 1 h. 2,4-Diamino-6-chloropyrimidine (**2**) (8.73 mmol) was added and stirred at room temperature for 11 h. The reaction solution was quenched with sat NH_4_Cl (50 mL) and extracted with EtOAc (150 mL × 3), and the combined organic layers dried with Na_2_SO_4_, filtered and concentrated. The residue was purified by column chromatography on silica gel using CH_2_Cl_2_/CH_3_OH (50:1, *v*/*v*) as the eluting solvent to give compounds **13a-d**.

*2,4-Diamino-6-(2-methoxyethoxy)pyrimidine* (**13a**). White solid 1.04 g, yield 65%. m.p. 152.9–153.9 °C; IR: υ_max_/cm^−1^ 3440 (NH), 3368 (NH), 1636 (C=N), 1563 (C=C), 1118 (C-O-C); ^1^H-NMR (DMSO-*d*_6_) δ 6.00 (s, 2H, N*H*_2_), 5.85 (s, 2H, N*H*_2_), 5.02 (s, 1H, Ar-*H*), 4.23–4.17 (m, 2H, C*H*_2_), 3.59–3.51 (m, 2H, C*H*_2_), 3.27 (s, 3H, C*H*_3_); ES-MS 185.1 (M + H)^+^; HRMS Calcd. for C_16_H_20_ClN_4_O_3_^+^ 185.1039, found 185.1040.

*2,4-Diamino-6-(3-methoxylpropoxy)pyrimidine* (**13b**). White solid 1.07 g, yield 62%. m.p. 104.2–104.8 °C; IR: υ_max_/cm^−1^ 3456 (NH), 3343 (NH), 1656 (C=N), 1572 (C=C), 1207 (C-O-C); ^1^H-NMR (DMSO-*d*_6_) δ 5.98 (s, 2H, N*H*_2_), 5.84 (s, 2H, N*H*_2_), 5.02 (s, 1H, Ar-*H*), 4.11 (t, *J* = 6.6, 2H, C*H*_2_), 3.40 (t, *J* = 6.3, 2H, C*H*_2_), 3.23 (s, 3H, C*H*_3_), 1.84 (m, 2H, C*H*_2_); ES-MS 199.1 (M + H)^+^; HRMS Calcd. for C_16_H_20_ClN_4_O_3_^+^ 199.1195, found 199.1198.

*2,4-Diamino-6-(thiazole-5-methoxy)pyrimidine* (**13c**). Yellow solid 1.44 g, yield 74%. m.p. 156.8–161.3 °C; IR: υ_max_/cm^−1^ 3466 (NH), 3320 (NH), 1625 (C=N), 1570 (C=C), 1454 (C=C), 1138 (C-O-C), 1061 (C-O-C); ^1^H-NMR (DMSO-*d*_6_) δ 9.06 (s, 1H, Ar-*H*), 7.97 (s, 1H, Ar-*H*), 6.07 (s, 2H, N*H*_2_), 5.98 (s, 2H, N*H*_2_), 5.44 (s, 2H, C*H*_2_), 5.04 (s, 1H, Ar-*H*); ES-MS 224.0 (M + H)^+^; HRMS Calcd. for C_16_H_20_ClN_4_O_3_^+^ 224.0606, found 224.0608.

*2,4-Diamino-6-(1-benzyl-1H-1,2,3-triazole-4-methoxy)pyrimidine* (**13d**). White solid 2.05 g, yield 79%. m.p.131.4–135.7 °C; IR: υ_max_/cm^−1^ 3421 (NH), 3333 (NH), 1631 (C=N), 1588 (N=N), 1451 (C=C), 1422 (C=C), 1197 (C-O-C); ^1^H-NMR (DMSO-*d*_6_) δ 8.20 (s, 1H, Ar-*H*), 7.36 (m, 5H, Ar-*H*), 6.04 (s, 2H, N*H*_2_), 5.96 (s, 2H, N*H*_2_), 5.59 (s, 2H, C*H*_2_), 5.20 (s, 2H, C*H*_2_), 5.02 (s, 1H, Ar-*H*); ES-MS 298.1 (M + H)^+^; HRMS Calcd. for C_16_H_20_ClN_4_O_3_^+^ 298.1416, found 298.1412.

#### General Procedure for the Synthesis of 2,4-Diamino-5-iodine-6-substitutedpyrimidine Derivatives **14a-d**

Under argon, to a solution of **13-d** (6.06 mmol) in dry CH_3_CN (35 mL) was added N-iodosuccinimide (9.09 mmol) and stirred at room temperature for 12 h. The reaction solution was extracted with EtOAc (150 mL × 3), and the combined organic layers were washed by NaHSO_3_, sat. NaHCO_3_ and sat. NaCl, and dried with Na_2_SO_4_, filtered and concentrated. The residue was purified by column chromatography on silica gel using CH_2_Cl_2_/CH_3_OH (80:1, *v*/*v*) as the eluting solvent to give compounds **14a-d**.

*2,4-Diamino-5-iodo-6-(2-methoxyethoxy)pyrimidine* (**14a**). Yellow solid 1.50 g, 80%. m.p. 109.7–111.0 °C; IR: υ_max_/cm^−1^ 3424 (NH), 3345 (NH), 1705 (C=N), 1554 (C=C), 1116 (C-O-C), 523 (C-I); ^1^H-NMR (DMSO-*d*_6_) δ 6.10 (s, 4H, N*H*_2_), 4.31–4.27 (m, 2H, C*H*_2_), 3.61–3.57 (m, 2H, C*H*_2_), 3.31 (s, 3H, C*H*_3_); ES-MS 310.9 (M + H)^+^; HRMS Calcd. for C_16_H_20_ClN_4_O_3_^+^ 311.0005, found 311.0003.

*2,4-Diamino-5-iodo-6-(3-methoxypropoxy)pyrimidine* (**14b**). Yellow solid 1.37 g, 70%. m.p. 124.0–125.8 °C; IR: υ_max_/cm^−1^ 3472 (NH), 3357 (NH), 1631 (C=N), 1553 (C=C), 1145 (C-O-C), 532 (C-I); ^1^H-NMR (DMSO-*d*_6_) δ 6.09 (s, 4H, N*H*_2_), 4.20 (t, *J* = 6.4, 2H, C*H*_2_), 3.45 (t, *J* = 6.3, 2H, C*H*_2_), 3.24 (s, 3H, C*H*_3_), 1.86 (m, 2H, C*H*_2_); ES-MS 325.0 (M + H)^+^; HRMS Calcd. for C_16_H_20_ClN_4_O_3_^+^ 325.0161, found 325.0159.

*2,4-Diamino-5-iodo-6-(thiazole-5-methoxy)pyrimidine* (**14c**). Yellow solid 1.90 g, yield 90%. m.p. 178.0–179.9 °C; IR: υ_max_/cm^−1^ 3436 (NH), 3305 (NH), 1624 (C=N), 1541 (C=C), 1442 (C=C), 1127 (C-O-C), 1103 (C-O-C), 545 (C-I); ^1^H-NMR (DMSO-*d*_6_) δ 9.08 (d, *J* = 0.5, 1H, Ar-*H*), 8.01 (d, *J* = 0.6, 1H, Ar-*H*), 6.24 (s, 4H, N*H*_2_), 5.51 (s, 2H, C*H*_2_); ES-MS 349.9 (M + H)^+^; HRMS Calcd. for C_16_H_20_ClN_4_O_3_^+^ 349.9572, found 349.9572.

*2,4-Diamino-5-iodo-6-(1-benzyl-1H-1,2,3-triazole-4-methoxy)pyrimidine* (**14d**). Yellow solid 2.38 g, yield 93%. m.p. 156.4–159.1 °C; IR: υ_max_/cm^−1^ 3441 (NH), 3340 (NH), 1618 (C=N), 1552 (N=N), 1446 (C=C), 1280 (C-O-C); ^1^H-NMR (DMSO-*d*_6_) δ 8.51 (s, 1H, Ar-*H*), 7.86–7.74 (m, 5H, Ar-*H*), 6.28 (s, 2H, N*H*_2_), 6.20 (s, 2H, N*H*_2_), 6.08 (s, 2H, C*H*_2_), 5.81 (s, 2H, C*H*_2_); ES-MS 424.0 (M + H)^+^; HRMS Calcd. for C_16_H_20_ClN_4_O_3_^+^ 424.0383, found 424.0380.

#### General Procedure for the Synthesis of 2,4-Diamino-5-aryl-6-substitutedpyrimidine Derivatives **16a-p**

(A) Under argon, to a mixed solution of EtOH/toluene/H_2_O (1:2:1, 60 mL) was added compounds **14a-d** (2.36 mmol), substituted phenylboronic acid **15** (3.55 mmol), Pd(dbpf)Cl_2_ (0.02 mmol) and K_2_CO_3_ (3.55 mmol) consecutively and then stirred at 90 °C for 24 h. The reaction solution was extracted with EtOAc (150 mL × 3), and the combined organic layers were washed by H_2_O and dried with Na_2_SO_4_, filtered and concentrated. The residue was purified by column chromatography on silica gel using CH_2_Cl_2_/CH_3_OH (80:1, *v*/*v*) as the eluting solvent to the desired compounds.

(B) In a pressure tube, to a mixed solution of THF/H_2_O (1:1, 50 mL) was added compounds **14a-d** (2.40 mmol), substituted phenylboronic acid **15** (3.60 mmol), Pd(dbpf)Cl_2_ (0.03 mmol) and K_2_CO_3_ (3.60 mmol) consecutively and then stirred at 70 °C for 20 h. The reaction solution was extracted with EtOAc (150 mL × 3), and the combined organic layers were washed by H_2_O and dried with Na_2_SO_4_, filtered and concentrated. The residue was purified by column chromatography on silica gel using CH_2_Cl_2_/CH_3_OH (60:1, *v*/*v*) as the eluting solvent to the desired compounds.

*2,4-Diamino-5-(4-trifluoromethoxyphenyl)-6-(2-methoxyethoxy)pyrimidine* (**16a**). Method A. White solid 0.60 g, yield 74%. m.p. 178.3–179.0 °C; IR: υ_max_/cm^−1^ 3477 (NH), 3349 (NH), 1619 (C=N), 1553 (C=C), 1486 (C=C), 1447 (C=C), 1275 (C-F), 1222 (C-O-C), 1143 (C-O-C); ^1^H-NMR (DMSO-*d*_6_) δ 7.38–7.29 (m, 4H, Ar-*H*), 6.04 (s, 2H, N*H*_2_), 5.67 (s, 2H, N*H*_2_), 4.28–4.23 (m, 2H, C*H*_2_), 3.52–3.46 (m, 2H, C*H*_2_), 3.18 (s, 3H, C*H*_3_); ES-MS 345.1 (M + H)^+^; HRMS Calcd. for C_16_H_20_ClN_4_O_3_^+^ 345.1175, found 345.1176.

*2,4-Diamino-5-(3-trifluoromethoxyphenyl)-6-(2-methoxyethoxy)pyrimidine* (**16b**). Method A. White solid 0.60 g, yield 74%. m.p. 80.1–81.7 °C; IR: υ_max_/cm^−1^ 3347 (NH), 3393 (NH), 1620 (C=N), 1561 (C=C), 1451 (C=C), 1292 (C-F), 1207 (C-O-C), 1157 (C-O-C); ^1^H-NMR (DMSO-*d*_6_) 7.49–7.44 (m, 1H, Ar-*H*), 7.29 (d, *J* = 7.8, 1H, Ar-*H*), 7.20 (d, *J* = 6.4, 2H, Ar-*H*), 6.08 (s, 2H, N*H*_2_), 5.73 (s, 2H, N*H*_2_), 4.28–4.23 (m, 2H, C*H*_2_), 3.51–3.46 (m, 2H, C*H*_2_), 3.18 (s, 3H, C*H*_3_); ES-MS 345.1 (M + H)^+^; HRMS Calcd. for C_16_H_20_ClN_4_O_3_^+^ 345.1175, found 345.1177.

*2,4-Diamino-5-[3-(2,2,2-trifluoroethoxymethyl)phenyl]-6-(2-methoxyethoxy)pyrimidine* (**16c**). Method A. Colorless oil 0.61 g, yield 70%; IR: υ_max_/cm^−1^ 3480 (NH), 3383 (NH), 1607 (C=N), 1558 (C=C), 1437 (C=C), 1279 (C-F), 1160 (C-O-C), 1121 (C-O-C); ^1^H-NMR (DMSO-*d*_6_) δ 7.40–7.34 (m, 1H, Ar-*H*), 7.25–7.17 (m, 3H, Ar-*H*), 6.01 (s, 2H, N*H*_2_), 5.57 (s, 2H, N*H*_2_), 4.65 (s, 2H, C*H*_2_), 4.27–4.22 (m, 2H, C*H*_2_), 4.09 (q, *J* = 9.4, 2H, C*H*_2_), 3.51–3.45 (m, 2H, C*H*_2_), 3.18 (s, 3H, C*H*_3_); ES-MS 373.1 (M + H)^+^; HRMS Calcd. for C_16_H_20_ClN_4_O_3_^+^ 373.1488, found 373.1486.

*2,4-Diamino-5-[3,5-dimethyl-4-(N-methoxyaminosulfonyl)phenyl]-6-(2-methoxyethoxy)pyrimidine* (**16d**). Method A. White solid 0.68 g, yield 73%. m.p. 167.3–171.4 °C; IR: υ_max_/cm^−1^ 3466 (NH), 3364 (NH), 1633 (C=N), 1587 (C=C), 1507 (NH), 1435 (C=C), 1157 (SO_2_), 1078 (SO_2_), 1014 (SO_2_); ^1^H-NMR (DMSO-*d*_6_) δ 10.26 (s, 1H, N*H*), 7.15 (s, 2H, Ar-*H*), 6.09 (s, 2H, N*H*_2_), 5.77 (s, 2H, N*H*_2_), 4.29–4.23 (m, 2H, C*H*_2_), 3.62 (s, 3H, C*H*_3_), 3.53–3.47 (m, 2H, C*H*_2_), 3.22 (s, 3H, C*H*_3_), 2.59 (s, 6H, C*H*_3_); ES-MS 398.1 (M + H)^+^; HRMS Calcd. for C_16_H_20_ClN_4_O_3_^+^ 398.1498, found 398.1497.

*2,4-Diamino-5-[4-(3-trifluoromethoxyanilinoformoxyl)phenyl)-6-(2-methoxyethoxy)pyrimidine* (**16e**). Method A. White solid 0.77 g, yield 70%. m.p. 96.50–100.1 °C; IR: υ_max_/cm^−1^ 3424 (NH), 3345 (NH), 1705 (C=N), 1554 (C=C), 1116 (C-O-C), 523 (C-I); ^1^H-NMR (DMSO-*d*_6_) δ 10.48 (d, *J* = 10.3, 1H, N*H*), 7.94 (dd, *J* = 17.0, 4.9, 4H, Ar-*H*), 7.49 (t, *J* = 8.2, 1H, Ar-*H*), 7.43 (d, *J* = 8.4, 2H, Ar-H), 7.09 (d, *J* = 8.3, 1H, Ar-*H*), 6.09 (s, 2H, N*H*_2_), 5.70 (s, 2H, N*H*_2_), 4.31–4.25 (m, 2H, C*H*_2_), 3.53–3.48 (m, 2H, C*H*_2_), 3.21 (s, 3H, C*H*_3_); ES-MS 464.1 (M + H)^+^; HRMS Calcd. for C_16_H_20_ClN_4_O_3_^+^ 464.4252, found 464.4250.

*2,4-Diamino-5-(4-trifluoromethoxyphenyl)-6-(3-methoxypropoxy)pyrimidine* (**16f**). Method A. White solid 0.63 g, yield 75%. m.p. 189.3–190.9 °C; IR: υ_max_/cm^−1^ 3471 (NH), 3365 (NH), 1632 (C=N), 1557 (C=C), 1451 (C=C), 1276 (C-F), 1207 (C-O-C), 1160 (C-O-C); ^1^H-NMR (DMSO-*d*_6_) δ 7.33 (m, 4H, Ar-*H*), 6.02 (s, 2H, N*H*_2_), 5.64 (s, 2H, N*H*_2_), 4.15 (t, *J* = 6.5, 2H, C*H*_2_), 3.27 (t, *J* = 6.3, 2H, C*H*_2_), 3.16 (s, 3H, C*H*_3_), 1.74 (m, 2H, C*H*_2_); ES-MS 359.1 (M + H)^+^; HRMS Calcd. for C_16_H_20_ClN_4_O_3_^+^ 359.1331, found 359.1333.

*2,4-Diamino-5-(3-trifluoromethoxyphenyl)-6-(3-methoxypropoxy)pyrimidine* (**16g**). Method A. White solid 0.79 g, yield 94%. m.p. 165.1–165.4 °C; IR: υ_max_/cm^−1^ 3470 (NH), 3364 (NH), 1633 (C=N), 1559 (C=C), 1454 (C=C), 1283 (C-F), 1205 (C-O-C), 1154 (C-O-C); ^1^H-NMR (DMSO-*d*_6_) δ 7.47 (t, *J* = 8.0, 1H, Ar-*H*), 7.27 (d, *J* = 7.8, 1H, Ar-*H*), 7.21 (dd, *J* = 8.3, 1.0, 1H, Ar-*H*), 7.17 (s, 1H, Ar-*H*), 6.06 (s, 2H, N*H*_2_), 5.70 (s, 2H, N*H*_2_), 4.15 (t, *J* = 6.4, 2H, C*H*_2_), 3.29 (t, *J* = 6.3, 2H, C*H*_2_), 3.17 (s, 3H, C*H*_3_), 1.74 (m, 2H, C*H*_2_); ES-MS 359.1 (M + H)^+^; HRMS Calcd. for C_16_H_20_ClN_4_O_3_^+^ 359.1331, found 359.1330.

*2,4-Diamino-5-(4-trifluoromethoxyphenyl)-6-(thiazole-5-methoxy)pyrimidine* (**16h**). Method A. Yellow solid 0.79 g, yield 87%. m.p. 188.3–189.6 °C; IR: υ_max_/cm^−1^ 3429 (NH), 3327 (NH), 1601 (C=N), 1556 (C=C), 1449 (C=C), 1245 (C-F), 1105 (C-O-C), 1025 (C-O-C); ^1^H-NMR (DMSO-*d*_6_) δ 9.03 (s, 1H, Ar-*H*), 7.91 (s, 1H, Ar-*H*), 7.30 (s, 4H, Ar-*H*), 6.16 (s, 2H, N*H*_2_), 5.75 (s, 2H, N*H*_2_), 5.45 (s, 2H, C*H*_2_); ES-MS 384.0 (M + H)^+^; HRMS Calcd. for C_16_H_20_ClN_4_O_3_^+^ 384.0742, found 384.0745.

*2,4-Diamino-5-(3-trifluoromethoxyphenyl)-6-(thiazole-5-methoxy)pyrimidine* (**16i**). Method A. Yellow solid 0.66 g, yield 73%. m.p. 142.8–143.2 °C; IR (KBr): υ_max_/cm^−1^ 3467 (NH), 3361 (NH), 1628 (C=N), 1556 (C=C), 1452 (C=C), 1261 (C-F), 1106 (C-O-C), 1042 (C-O-C); ^1^H-NMR (DMSO-*d*_6_) δ 9.01 (s, 1H, Ar-*H*), 7.87 (s, 1H, Ar-*H*), 7.50–7.45 (m, 1H, Ar-*H*), 7.30 (d, *J* = 7.8, 1H, Ar-*H*), 7.21 (d, *J* = 6.4, 2H, Ar-*H*), 6.20 (s, 2H, N*H*_2_), 5.79 (s, 2H, N*H*_2_), 5.45 (s, 2H, C*H*_2_); ES-MS 384.0 (M + H)^+^; HRMS Calcd. for C_16_H_20_ClN_4_O_3_^+^ 384.0742, found 384.0740.

*2,4-Diamino-5-[3-(2,2,2-trifluoroethoxymethyl)phenyl]-6-(thiazole-5-methoxy)pyrimidine* (**16j**). Method A. Yellow solid 0.88 g, yield 91%. m.p. 126.3–129.0 °C; IR: υ_max_/cm^−1^ 3478 (NH), 3316 (NH), 1624 (C=N), 1553 (C=C), 1442 (C=C), 1282 (C-F), 1158 (C-O-C); ^1^H-NMR (DMSO-*d*_6_) δ 9.02 (s, 1H, Ar-*H*), 7.90 (s, 1H, Ar-*H*), 7.35 (t, *J* = 7.6, 1H, Ar-*H*), 7.21 (d, *J* = 7.7 , 1H, Ar-*H*), 7.14 (d, *J* = 8.6, 2H, Ar-*H*), 6.12 (s, 2H, N*H*_2_), 5.64 (s, 2H, N*H*_2_), 5.44 (s, 2H, C*H*_2_), 4.64 (s, 2H, C*H*_2_), 4.08 (q, *J* = 9.4, 2H, C*H*_2_); ES-MS 412.1 (M + H)^+^; HRMS Calcd. for C_16_H_20_ClN_4_O_3_^+^ 412.1055, found 412.1056.

*2,4-Diamino-5-[3,5-dimethyl-4-(N-methoxyaminosulfonyl)phenyl]-6-(thiazole-5-methoxy)pyrimidine* (**16k**). Method A. White solid 0.74 g, yield 72%. m.p. 139.2–142.6 °C; IR: υ_max_/cm^−1^ 3463 (NH), 3358 (NH), 1616 (C=N), 1589 (C=C), 1490 (C=C), 1448 (C=C), 1168 (SO_2_), 1076 (SO_2_), 1009 (SO_2_); ^1^H-NMR (DMSO-*d*_6_) δ 10.26 (s, 1H, N*H*), 9.05 (s, 1H, Ar-*H*), 7.92 (s, 1H, Ar-*H*), 7.07 (s, 2H, Ar-*H*), 6.21 (s, 2H, N*H*_2_), 5.84 (s, 2H, N*H*_2_), 5.46 (s, 2H, C*H*_2_), 3.61 (s, 3H, C*H*_3_), 2.57 (s, 6H, C*H*_3_); ES-MS 437.1 (M + H)^+^; HRMS Calcd. for C_16_H_20_ClN_4_O_3_^+^ 437.1066, found 437.1065.

*2,4-Diamino-5-[4-(3-trifluoromethoxyanilinocarbonyl)phenyl]-6-(thiazole-5-methoxy)pyrimidine* (**16l**). Method A. Yellow solid 1.17 g, yield 99%. m.p. 180.9–182.9 °C; IR: υ_max_/cm^−1^ 3430 (NH), 3340 (NH), 3200 (NH), 1654 (C=O), 1608 (C=N), 1551 (C=C), 1484 (C=C), 1442 (C=C), 1257 (C-F); ^1^H-NMR (DMSO-*d*_6_) δ 10.46 (s, 1H, N*H*), 9.04 (s, 1H, Ar-*H*), 7.94 (d, *J* = 8.6, 4H, Ar-*H*), 7.79 (d, *J* = 8.2, 1H, Ar-*H*), 7.48 (t, *J* = 8.2, 1H, Ar-*H*), 7.36 (d, *J* = 8.4, 2H, Ar-*H*), 7.09 (d, *J* = 8.2, 1H, Ar-*H*), 6.20 (s, 2H, N*H*_2_), 5.77 (s, 2H, N*H*_2_), 5.47 (s, 2H, C*H*_2_); ES-MS 503.1 (M + H)^+^; HRMS Calcd. for C_16_H_20_ClN_4_O_3_^+^ 503.1113, found 503.1111.

*2,4-Diamino-5-(4-trifluoromethoxyphenyl)-6-(1-benzyl-1H-1,2,3-triazole-4-methoxy)pyrimidine* (**16m**). Method A. White solid 0.98 g, yield 91%. m.p.159.5–160.6 °C; IR: υ_max_/cm^−1^ 3489 (NH), 3371 (NH), 1608 (C=N), 1556 (N=N), 1492 (C=C), 1439 (C=C), 1261 (C-O-C), 1219 (C-F); ^1^H-NMR (DMSO-*d*_6_) δ 8.15 (s,1H, Ar-*H*), 7.40–7.23 (m, 9H, Ar-*H*), 6.17 (s, 2H, N*H*_2_), 5.73 (s, 2H, N*H*_2_), 5.57 (s, 2H, C*H*_2_), 5.24 (s, 2H, C*H*_2_); ES-MS 458.1 (M + H)^+^; HRMS Calcd. for C_16_H_20_ClN_4_O_3_^+^ 458.1552, found 458.1555.

*2,4-Diamino-5-(3-trifluoromethoxyphenyl)-6-(1-benzyl-1H-1,2,3-triazole-4-methoxy)pyrimidine* (**16n**). Method A. White solid 0.98 g, yield 91%. m.p. 171.9–173.0 °C; IR: υ_max_/cm^−1^ 3495 (NH), 3376 (NH), 1607 (C=N), 1556 (N=N), 1491 (C=C), 1431 (C=C), 1278 (C-O-C), 1219 (C-F); ^1^H-NMR (DMSO-*d*_6_) δ 8.15 (s, 1H, Ar-*H*), 7.40–7.23 (m, 9H, Ar-*H*), 6.17 (s, 2H, N*H*_2_), 5.73 (s, 2H, N*H*_2_), 5.57 (s, 2H, C*H*_2_), 5.24 (s, 2H, C*H*_2_); ES-MS 458.1 (M + H)^+^; HRMS Calcd. for C_16_H_20_ClN_4_O_3_^+^ 458.1552, found 458.1553.

*2,4-Diamino-5-(4-methoxycarbonylphenyl)-6-(1-benzyl-1H-1,2,3-triazole-4-methoxy)pyrimidine* (**16o**). Method B. Gray solid 0.70 g, yield 68%. m.p. 218.6–222.3 °C; IR: υ_max_/cm^−1^ 3487 (NH), 3440 (NH), 1709 (C=O), 1616 (C=N), 1555 (N=N), 1483 (C=C), 1440 (C=C), 1285 (C-O-C); ^1^H-NMR (DMSO-*d*_6_): 8.16 (s, 1H, Ar-*H*), 7.87 (d, *J* = 8.4, 2H, Ar-*H*), 7.39–7.25 (m, 7H, Ar-*H*), 6.20 (s, 2H, N*H*_2_), 5.77 (s, 2H, N*H*_2_), 5.57 (s, 2H, C*H*_2_), 5.24 (s, 2H, C*H*_2_), 3.86 (s, 3H, C*H*_3_); ES-MS 432.1 (M + H)^+^; HRMS Calcd. for C_16_H_20_ClN_4_O_3_^+^ 432.1784, found 432.1786.

*2,4-Diamino-5-(3-methoxycarbonylphenyl)-6-(1-benzyl-1H-1,2,3-triazole-4-methoxy)pyrimidine* (**16p**). Method B. Yellow solid 0.53 g, yield 51%. m.p. 210.4–213.7 °C; IR: υ_max_/cm^−1^ 3432 (NH), 3351 (NH), 1706 (C=O), 1619 (C=N), 1564 (N=N), 1485 (C=C), 1442 (C=C), 1252 (C-O-C); ^1^H-NMR (DMSO-*d*_6_) δ 8.13 (s, 1H, Ar-*H*), 7.80 (dd, *J* = 8.8, 4.4, 2H, Ar-*H*), 7.44 (d, *J* = 5.1, 2H, Ar-*H*), 7.34 (dd, *J* = 9.1, 6.9, 3H, Ar-*H*), 7.28–7.24 (m, 2H, Ar-*H*), 6.16 (s, 2H, N*H*_2_), 5.71 (s, 2H, N*H*_2_), 5.56 (s, 2H, C*H*_2_), 5.24 (s, 2H, C*H*_2_), 3.82 (s, 3H, C*H*_3_); ES-MS 432.1 (M + H)^+^; HRMS Calcd. for C_16_H_20_ClN_4_O_3_^+^ 432.1784, found 432.1785.

### 3.3. Determination of Anti-Mycobacterial Activity

The minimum inhibitory concentrations (MIC) and minimum bactericidal concentrations (MBC) of the test compounds were determined as described previously [19] with minor modifications. In brief, the MIC was determined using the microbroth dilution method in 96-well microtitre plates. *Mycobacterium tuberculosis* H37Ra (ATCC 25177) was used as the test strain, at a final inoculum of approximately 10^5^ cfu/mL. The compounds were dissolved in DMSO and diluted in 1% DMSO in 7H9 broth to obtain concentrations ranging from 100 μg/mL to 1.56 μg/mL. All inoculated plates were sealed with Parafilm and incubated at 36 °C for 28 days. On days 14 and 28, all wells were observed for visible growth, and 10 μL was removed from each well for subculturing on compound-free 7H10 agar plates which were incubated for six weeks at 36 °C. For each compound, the lowest concentration to inhibit the growth of H37Ra in broth culture was taken to be the MIC. The MBC was the lowest concentration to inhibit the formation of colonies on the agar subculture, up to six weeks of incubation.

### 3.4. Molecular Docking

The crystal structure of *mt*-DHFR in complex with NADPH and MTX (PDB ID: 1DF7) [20] was used as the receptor for molecular docking, which was performed by *GOLD Docking:* GOLD (v 5.2.2 Genetic Optimization for Ligand Docking) [21]. The bound ligand, MTX was used as a reference to indicate the binding site, and each molecule was docked 10 times with the default automatic genetic algorithm parameter settings and the results were evaluated by Gold Score.

### 3.5. Molecular Dynamic Simulation

The geometry of the docked ligand was optimized by using the B3LYP 6–31 G* basis set within Gaussian09 [22], and the atom-centered point charges were calculated to fit the electrostatic potential using RESP [23]. The parameters of NADPH were obtained from AMBER parameter database (http://www.pharmacy.manchester.ac.uk/bryce/amber/, uploaded by U. Ryde).

The docked compound and *mt*-DHFR complex was explicitly solvated in a truncated octahedral box of TIP3P model water (at least 12 Å from the complex to avoid periodic artifacts from occurring) by using Amber 16 with the amber ff14SB force field. The system charges were neutralized by adding enough *K*^+^ ions by using the tleap module (AmberTools 16). The explicit solvent models and the NPT ensemble (T = 300 K; P = 1 atm) were performed on all molecular dynamic simulations. Periodic boundary conditions (PBC) and particle-mesh-Ewald method (PME) [24] were used to treat long-range electrostatic effects, with the temperature coupled to an external bath using a weak coupling algorithm [25]. The non-bonded interaction cutoff was set as 8 Å. The bond interactions involving H-atoms were constrained by using the SHAKE algorithm. The time step necessary to solve the Newton’s equations was chosen to be equal to 2 fs and the trajectory files were collected every 10 ps. All trajectory analysis was performed with the Ptraj module in the AmberTools 16 and examined visually using VMD software [26].

The whole system was first optimized by energy minimization, followed by 110 ns molecular dynamic simulation, including 10 ns equilibration and 100 ns production simulations. The last 80 ns (1000 snapshots, 80 ps intervals) stable, equilibrated trajectories of the production simulation were taken for binding free energy calculations performed by using MM-PBSA [27,28,29] (included in AMBERTOOLS 16).

The entropy was estimated by using the Normal Mode program [28,29] within the AMBER16 suite. Because the entropy calculations are computationally intensive, only 100 snapshots from the last 80 ns trajectories were used for the normal-mode analysis.

## 4. Conclusions

In conclusion, in order to occupy the GOL binding site on *mt*-DHFR and maintain proper hydrophobicities to allow the compounds to function in the *Mtb* whole cell assay, we designed and synthesized three series of compounds which contain a hydrophobic side chain. Among them, the compounds with a thiazole side chain significantly inhibited the growth of *Mtb*, and the best inhibition effect was observed on compound **16l**. More interestingly, this compound showed selectivity for *Mtb* over vero cells, which makes it potentially useful as a lead compound for future studies on anti-TB drugs. Unfortunately, we are currently unable to confirm **16l** as an *mt*-DHFR inhibitor by performing the binding assay on the pure *mt*-DHFR enzyme.

Sulfonamides were the first anti-bacterial agents to be used for the treatment of tuberculosis [30]. They were subsequently replaced by the standard first-line anti-TB drugs rifampicin, isoniazid, ethambutol and pyrazinamide, which had greater anti-TB activity. With the increase of multidrug resistant TB in the past two decades, and the therapeutic failures with standard anti-TB drugs, respiratory physicians have to resort the treatment with other antibiotics and chemotherapeutic agents including the trimethoprim-sulfamethoxazole combination [31]. While sulfamethoxazole competes with *para*-aminobenzoic acid (PABA) for the enzyme dihydropteroate synthetase, trimethoprim directly inhibits the dihydrofolate reductase for the reduction of dihydrofolic acid to tetrahydrofolic acid. This combination was found to have in vitro activity for up to 98% of *Mtb* isolates [32]. However, both drugs are bacteriostatic, and are associated with drug resistance. Sulfonamide resistance has been reported to result from a point mutation or the acquisition of a plasmid which enables the synthesis of a dihydropteroic synthetase that has poor affinity for sulfonamides. Similarly, trimethoprim can be rendered ineffective by plasmid- and transposon-mediated production of an altered dihydrofolate reductase that has markedly reduced affinity for the drug. In some studies, trimethoprim, when used by itself, has been shown to have little activity on some strains of *Mtb* [33]. The new compounds we synthesized appear to be bactericidal against *Mtb*, making it a better drug for TB therapy in immunocompromised patients. Further studies are required to test for the frequency of mutation to resistance among clinical strains of *Mtb* and whether the compounds can be used in combination with other anti-TB drugs or antibiotics for synergistic activity or to retard the development of resistance.

## Figures and Tables

**Table 1:**
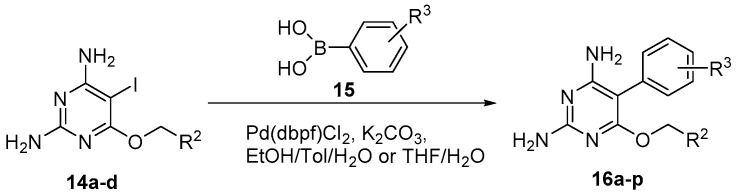
Synthesis of 2,4-diamino-5-aryl-6-substituted pyrimidine derivatives **16a-p** via Suzuki reactions.

Entry	R^2^	R^3^	Product	Yield *^c^* (%)	Clog P *^d^*
1	-CH_2_OCH_3_ (**14a**)	4-OCF_3_	**16a** *^a^*	74	2.99
2	-CH_2_OCH_3_ (**14a**)	3-OCF_3_	**16b** *^a^*	74	2.99
3	-CH_2_OCH_3_ (**14a**)	3-CH_2_OCH_2_CF_3_	**16c** *^a^*	70	2.29
4	-CH_2_OCH_3_ (**14a**)	3-CH_3_-5-CH_3_-4-SO_2_NHOCH_3_	**16d** *^a^*	73	1.19
5	-CH_2_OCH_3_ (**14a**)	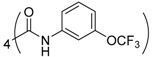	**16e** *^a^*	70	3.59
6	-CH_2_CH_2_OCH_3_ (**14b**)	4-OCF_3_	**16f** *^a^*	75	3.37
7	-CH_2_CH_2_OCH_3_ (**14b**)	3-OCF_3_	**16g** *^a^*	94	3.37
8	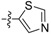 (**14c**)	4-OCF_3_	**16h** *^a^*	87	2.81
9	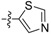 (**14c**)	3-OCF_3_	**16i** *^a^*	73	2.81
10	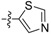 (**14c**)	3-CH_2_OCH_2_CF_3_	**16j** *^a^*	91	2.11
11	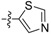 (**14c**)	3-CH_3_-5-CH_3_-4-SO_2_NHOCH_3_	**16k** *^a^*	72	1.01
12	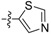 (**14c**)	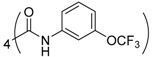	**16l** *^a^*	99	3.42
13	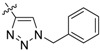 (**14d**)	4-OCF_3_	**16m** *^a^*	91	3.56
14	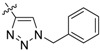 (**14d**)	3-OCF_3_	**16n** *^a^*	91	3.56
15	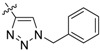 (**14d**)	4-COOCH_3_	**16o** *^b^*	68	2.39
16	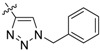 (**14d**)	3-COOCH_3_	**16p** *^b^*	51	2.39

Reaction Conditions: *^a^* Pd(dbpf)Cl_2_ (0.02 mmol) and K_2_CO_3_ in EtOH/toluene/H_2_O at 90 °C for 24 h; *^b^* Pd(dbpf)Cl_2_ (0.02 mmol) and K_2_CO_3_ in THF/H_2_O at 70 °C for 20 h in sealed tube; *^c^* Isolated yields; *^d^* Calculated using ChemBioDraw (PerkinElmer, Waltham, MA, USA) 12.0.

**Table 2:**
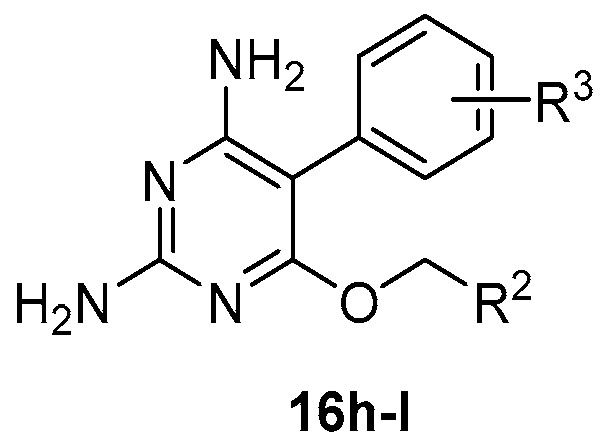
Minimum inhibitory concentrations (MIC) and minimum bactericidal concentrations (MBC) of compounds **16h-l** showing anti-tubercular activity.

Compound	R^2^	R^3^	MIC/MBC (μg/mL)
**16h**	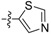	4-OCF_3_	25/25
**16i**	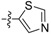	3-OCF_3_	50/50
**16j**	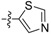	3-CH_2_OCH_2_CF_3_	25/25
**16k**	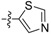	3-CH_3_-5-CH_3_-4-SO_2_NHOCH_3_	100/100
**16l**	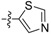	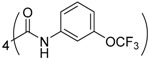	6.25/12.5
Rifampicin			0.313/0.313

**Table 3:** Binding free energies (Kcal/mol) of compound **16l** in *mt*-DHFR.

Simulations	∆E_vdw_	∆E_ele_	∆G_pb_	∆G_np_	∆G_gas_	∆G_solv_	∆G_mmpbsa_	T∆S	∆G_binding_
**16l**	−42.37 ± 0.14	−18.16 ± 0.24	37.55 ± 0.33	−5.62 ± 0.01	−60.53 ± 0.30	31.93 ± 0.32	−28.60 ± 0.18	−25.13 ± 0.73	−3.47
